# Plasma‐based analysis of *ERBB2* mutational status by multiplex digital PCR in a large series of patients with metastatic breast cancer

**DOI:** 10.1002/1878-0261.13592

**Published:** 2024-01-29

**Authors:** Julien Corné, Véronique Quillien, Florence Godey, Mathilde Cherel, Agathe Cochet, Fanny Le Du, Lucie Robert, Héloïse Bourien, Angélique Brunot, Laurence Crouzet, Christophe Perrin, Claudia Lefeuvre‐Plesse, Véronique Diéras, Thibault De la Motte Rouge

**Affiliations:** ^1^ Department of Biology Centre Eugène Marquis, Unicancer Rennes France; ^2^ Department of Medical Oncology Centre Eugène Marquis, Unicancer Rennes France; ^3^ INSERM U1242 University of Rennes France

**Keywords:** circulating tumor DNA, *ERBB2*, liquid biopsies, metastatic breast cancer, multiplex digital PCR, plasma

## Abstract

Erb‐b2 receptor tyrosine kinase 2 (*ERBB2*)‐activating mutations are therapeutically actionable alterations found in various cancers, including metastatic breast cancer (MBC). We developed multiplex digital PCR assays to detect and quantify *ERBB2* mutations in circulating tumor DNA from liquid biopsies. We studied the plasma from 272 patients with hormone‐receptor‐positive, human epidermal growth factor receptor 2‐negative (HR+/HER2−) MBC to detect 17 *ERBB2* mutations using a screening assay. The assay was developed on the three‐color Crystal dPCR™ naica® platform with a two‐step strategy for precise mutation identification. We found that nine patients (3.3%) harbored at least one *ERBB2* mutation. The mutation rate was higher in patients with lobular histology (5.9%) compared to invasive breast carcinoma of no special type (2.6%). A total of 12 mutations were found with the following frequencies: L755S (25.00%), V777L (25.00%), S310Y (16.67%), L869R (16.67%), S310F (8.33%), and D769H (8.33%). Matched tumor samples from six patients identified the same mutations with an 83% concordance rate. In summary, our highly sensitive multiplex digital PCR assays are well suited for plasma‐based monitoring of *ERBB2* mutational status in patients with MBC.

## Graphical abstract

Erb‐b2 receptor tyrosine kinase 2 (*ERBB2*)‐activating mutations are therapeutically actionable alterations found in various cancers, including metastatic breast cancer (MBC). We developed highly sensitive multiplex digital PCR assays to detect and quantify *ERBB2* mutations in circulating tumor DNA from liquid biopsies and found mutations in 3.3% of 272 patients with hormone‐receptor‐positive, human epidermal growth factor receptor 2‐negative (HR+/HER2−) MBC.

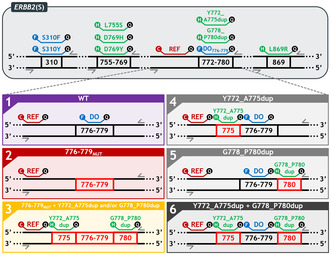

**Abbreviations**
cfDNA: circulating cell‐free DNA
COSMIC: Catalogue of Somatic Mutations in Cancer
ctDNA: circulating tumor DNA
CV: coefficient of variation
DO: drop‐off
dPCR: digital PCR
ER: estrogen receptor
ERBB2: Erb‐b2 receptor tyrosine kinase 2
ESCAT: ESMO Scale for Clinical Actionability of Molecular Targets
ET: endocrine therapy
FFPE: formalin‐fixed, paraffin‐embedded
gBlock: gBlock gene fragments
gDNA: genomic DNA
HER2: human epidermal growth factor receptor 2
HGVS: Human Genome Variation Society
HR: hormone receptor
IBC‐NST: invasive breast carcinoma of no special type
IDC: invasive ductal carcinoma
ILC: invasive lobular carcinoma
LOB: limit of blank
LOD: limit of detection
MAF: mutant allelic frequency
MBC: metastatic breast cancer
MUT: mutated
NF1: neurofibromin 1
NGS: next‐generation sequencing
OS: overall survival
PIK3CA: phosphatidylinositol‐4,5‐bisphosphate 3‐kinase catalytic subunit alpha
REF: reference
SD: stable disease
WT: wild‐type

## 1 Introduction

Human epidermal growth factor receptor 2 (HER2) is encoded by the Erb‐b2 receptor tyrosine kinase 2 (*ERBB2*) gene. HER2 is a common oncogenic driver in breast cancer and a major drug target. Most deregulation of HER2 signaling is caused by HER2/*ERBB2* amplification but activating mutations leading to constitutive HER2 signaling can also occur, both in patients with hormone receptor (HR)‐positive, HER2‐negative or HR+/HER2+ metastatic breast cancer (MBC), with different mutation landscapes linked to different therapeutic pressure on the intracellular and extracellular domain of the HER2 protein [1].

*ERBB2* mutations are rare in primary breast tumors. In a large series of 2433 tumors, 2.9% of samples were found mutated, with a similar proportion for estrogen receptor (ER)‐positive (2.8%) and ER‐negative (3.2%) tumors [2]. Similar proportions (between 2% and 3%) are found using online databases [3]. Further, a series of 5605 recurrent MBC with HR and HER2 status not specified found 138 mutated tumors (2.4%) [4].

The percentage of tumors harboring *ERBB2* mutation could be higher at the metastatic stage of the disease; it is between 3.2% and 4.2% in online databases [3], was 3% in a series of 381 HR+/HER2− patients [5], and was up to 7% in a series of 169 patients with ER‐positive MBC previously treated with endocrine therapy (ET) [6]. These mutations could, therefore, be acquired under the selective pressure of hormone therapy. A large series of 1501 HR+/HER2− tumors corroborated this; the *ERBB2* and neurofibromin 1 (*NF1*) genes had the greatest difference in mutational frequency between pre‐ and post‐hormonal therapy alongside expected *ESR1* mutations (a common cause of acquired resistance to certain types of ET) [7]. *ERBB2* and *ESR1* mutations have been reported as mutually exclusive [3, 7, 8, 9]. *ERBB2* mutation could, therefore, be an alternative mechanism of ET‐acquired resistance.

The positivity rate may differ according to the breast cancer's histologic type. Invasive lobular carcinoma (ILC) is the second most common type of breast cancer after invasive breast carcinoma of no special type (IBC‐NST). *ERBB2* was mutated in 5.1% of samples in 630 patients with primary ILC [10]. Similar results have been reported in smaller ILC series; 5% (series with *n* = 106 patients) [11], 4.3% (*n* = 144 patients) [12], and 4% (*n* = 127 patients) compared to 1.4% (*n* = 490 patients with IBC‐NST), a statistically significant difference [13]. In the metastatic stage, this proportion also increases; after pooling patients with ER‐positive ILC MBC of METABRIC (EGAS00000000083) and MSK/IMPACT (NCT01775072) studies, 43 patients out of 398 (10.8%) had *ERBB2* mutations. The prevalence of *ERBB2* mutation was 2.7% for the 2137 ER‐positive IBC‐NST metastatic patients [14].

Studies have investigated a possible link between *ERBB2* mutations and patient prognosis. In a retrospective case–control study of patients with HR+/HER2− MBC, a higher prevalence of bone and liver metastases was observed in *ERBB2*‐mutated cancers; this was not associated with a worse prognosis in terms of overall survival (OS) from metastatic disease [9]. For the 2509 patients with primary or MBC of the METABRIC cohort, the presence of all *ERBB2* mutations taken together was not associated with reduced OS. On the other hand, in the subgroup of ILC patients, all mutations and selected activating mutations (L755S and exon 20 ins/del) were associated with significantly worse OS; this suggests that *ERBB2* mutations induce earlier relapse in ILC than IBC‐NST patients [14].

*ERBB2*‐activating mutations are therapeutically actionable alterations. The mutations are classified as ESCAT (ESMO Scale for Clinical Actionability of Molecular Targets) level II, or “investigational targets that likely define a patient population that benefits from a targeted drug but additional data are needed” [15]. Several HER2 tyrosine kinase inhibitors have been developed and tested in pre‐clinical models and clinical trials [16]. In the SUMMIT basket study (NCT01953926), patients with *ERBB2*‐mutant tumors were treated with neratinib, a pan‐HER tyrosine kinase inhibitor. The drug exhibited the greatest degree of activity in the 25 breast cancer patients (compared to 125 other advanced‐stage solid tumors) [17]. In another cohort of the amended protocol, progression‐free survival, while modest, was longer in ER‐positive patients receiving neratinib in combination with fulvestrant (a selective ER degrader) [18].

Other trials have supported these findings. The mutHER trial (NCT01670877) evaluated neratinib plus fulvestrant in patients with ER+/*ERBB2*mut (without amplification) MBC. The clinical benefit rates were 38% for fulvestrant previously treated and 30% for fulvestrant‐naive patients, and patients with lobular histology were particularly responsive to the treatment [19]. The PlasmaMATCH trial (NCT0318263) assessed the ability of circulating tumor DNA (ctDNA) testing to select patients for mutation‐directed therapy. Nine out of the 20 patients (45%) with *ERBB2* mutation had a clinical benefit from neratinib. There was a 100% concordance between digital PCR (dPCR) ctDNA testing and tissue sequencing for 39 contemporaneous paired samples [20]. Other pan‐HER tyrosine kinase inhibitors (e.g., poziotinib and pyrotinib) are being tested in advanced breast cancer and other advanced solid tumors.

Given their therapeutic potential, detection of these mutations is of clinical interest for patients, and it is more appropriate to detect them at the metastatic stage of the disease, according to the literature. Obtaining a metastatic tissue biopsy can be challenging for the clinician or uncomfortable for the patients. Fortunately, several studies in patients with MBC have shown that genomic alterations found in circulating cell‐free DNA (cfDNA) predict treatment responses at least as reliably as tumor analysis [20].

We have developed multiplex assays on the three‐color Crystal dPCR™ naica® platform (Stilla Technologies, Villejuif, France) to detect *ERBB2* mutations in the plasma of patients with MBC. Our screening test detects the 10 *ERBB2* mutations most frequently observed in breast cancer (p.S310F, p.S310Y, p.L755S, p.D769H, p.D769Y, p.G776V, p.V777L (G>C), p.V777L (G>T), p.V777M, and p.L869R) according to the Catalog of Somatic Mutations in Cancer (COSMIC) database. It also detects p.G778_S779insLPG and p.G778_S779insLPS, which accounted for around 5% of all the detected mutations in one study [7]. Less frequent mutations (such as p.G778_P780dup) located between codons 776 and 779 were also detected with the drop‐off system used at this location. We present the analytical performance of this screening assay, our strategy to identify the *ERBB2* mutation(s) in positive cases, and the results of a large series obtained from patients with HR+/HER2− MBC.

## 2 Materials and methods

### 2.1 Patients

A total of 304 plasma samples from 272 women with HR+/HER2− MBC treated at the Department of Medical Oncology of the Centre Eugène Marquis in Rennes were examined (result details in Dataset S1). A blood sample was prospectively collected (between February 2017 and April 2023) from each patient at the time of disease progression for cfDNA extraction. The research study and protocols were conducted under French legal guidelines and fulfilled the requirements of the local institutional ethics committee (CREDO, license number: RT‐159). Written informed consent was obtained from all patients. The study methodologies conformed to the standards set by the Declaration of Helsinki.

### 2.2 Sample collection and processing

For cfDNA samples, 20 mL of blood was collected using two 10 mL K_2_EDTA blood collection tubes (BD Vacutainer®; Beckton, Dickinson, Franklin Lakes, NJ, USA) and processed within 4 h of collection. Plasma was obtained through double centrifugation at 1600 ***g*** for 15 min and 4500 ***g*** for 10 min. Samples were stored at −80 °C prior to cfDNA extraction.

Tumor samples were either frozen samples obtained from the processing of biological samples through the Centre de Ressources Biologiques (CRB)–Santé of Rennes BB‐0033‐00056 (http://www.crbsante‐rennes.com) or formalin‐fixed, paraffin‐embedded (FFPE) samples that had been used for the histopathological diagnostic and stored in the Ouest Pathologie Anatomopathology Laboratory (Rennes), which also performed the quality control estimates of tumor cells percentages.

### 2.3 Nucleic acid extractions, quality, and quantity assessments

As described previously [21, 22], cfDNA samples were extracted from 1.5 to 5 mL of plasma using the QIAamp Circulating Nucleic Acid kit (Qiagen, Hilden, Germany) and were resuspended in a final volume of 50 μL of AVE buffer. Frozen tumor genomic DNA (gDNA) samples were extracted from two freshly cut 10‐μm sections using the QIAamp DNA Mini kit (Qiagen) and were resuspended in a final volume of 100 μL of AE buffer. FFPE tumor gDNA samples were extracted from two freshly cut 10 ‐μm sections using the QIAamp DNA FFPE Tissue kit (Qiagen) and were resuspended in a final volume of 100 μL of ATE buffer.

Formalin‐fixed, paraffin‐embedded DNA samples were treated with 0.5 U of uracil‐DNA glycosylase (Thermo Fisher Scientific, Waltham, MA, USA) at 50 °C for 30 min prior to dPCR testing as in [23] to improve the quality of the results. The quality of the extracted nucleic acids was assessed using the High Sensitivity DNA kit on a 2100 Bioanalyzer Instrument (Agilent Technologies, Santa Clara, CA, USA). The quantity of the extracted nucleic acids was assessed using the Qubit™ dsDNA HS Assay kit on a Qubit™ 3.0 Fluorometer (Thermo Fisher Scientific) (Table S1).

### 2.4 *In silico* design and verifications of the *ERBB2* assays

All primers and hydrolysis probes (Table S2) were designed as previously described [21, 22], using the sequence of the *ERBB2* gene (NG_007503.1) for the studied mutations (Table S3). All oligonucleotides were synthesized by Eurogentec (Seraing, Belgium). The *ERBB2* screening assay was designed to cover as many pathogenic mutations as possible in a single multiplex assay with the best possible coverage rate in terms of mutation frequencies. It covers all pathogenic *ERRB2* mutations identified for breast carcinoma tumor samples with a frequency > 2% in the COSMIC database.

### 2.5 Design of *ERBB2*‐mutated cfDNA‐like positive controls

We designed gBlock® Gene Fragments (gBlock) (Integrated DNA Technologies, Coralville, IA, USA) of 166 bp for use as positive controls (Table S4). We selected the six most frequent mutations occurring on codons 776–779 for the Drop‐Off_776–779_ system; these mutations represent 99% of all of the pathogenic and non‐pathogenic mutations identified on this hotspot for breast carcinoma tumor samples in the COSMIC database (Fig. S1).

### 2.6 Other sources of DNA used during validation experiments

Wild‐type (WT) gDNA were extracted from the peripheral blood mononuclear cells of three healthy donors.

### 2.7 Digital PCR workflow and crystal dPCR™ data analysis

All dPCR experiments were performed and analyzed following the workflow detailed in [21, 22]. Briefly, each PCR was performed in a final volume of 25 μL, containing 5 μL of 5× naica® multiplex PCR MIX Buffer A (Stilla Technologies), 1 μL of naica® multiplex PCR MIX Buffer B (Stilla Technologies), 2.5 μL of homemade 10× *ERBB2* assay (Table S2), 1.5 μL of DNase/RNase Free UltraPure™ distilled water (Invitrogen™, Thermo Fisher Scientific), and 15 μL of input cfDNA sample. Highly concentrated samples were diluted in nuclease‐free water to reach a maximum theoretical concentration of 10 000 copies/PCR (33 ng/PCR) to limit background noises. Additionally, samples with low concentrations were assayed in two or three replicates to increase sensitivity by investigating at least 10 ng/PCR. A negative H_2_O control and a positive control containing a mixture of WT gDNA and mutated (MUT) gBlocks were included in each run. Each PCR program included an initial “partition” step that allowed for the formation of 12 900–25 800 droplets of ~0.68 nL, self‐arranged into a crystal‐like pattern. This step was followed by PCR amplification cycles (Table S5).

The chips were imaged with the naica® Prism3 scanner using the crystal reader™ software v2.4.0.3 (Stilla Technologies) (Table S6), and the analyses were performed using crystal miner™ software v2.4.0.3 (Stilla Technologies). Notably, thresholds for classifying WT and MUT droplet clusters for patient samples were set manually using the polygon gates on 2D dot plots based on the positions of the positive control clusters. These results were then LOB‐corrected to account for the potential presence of false‐positive droplets using the equation previously presented [21, 22].

### 2.8 Determination of the limits of blank (LOB_95%_) and the theoretical limits of detection (LOD_95%_)

Determination of the limits of blank (LOB_95%_), defined as the maximum number of false‐positive droplets expected in a chamber at a probability of 95% (i.e., an α risk equal to 5%) in a sample containing no target sequence, and the theoretical limits of detection (LOD_95%_), defined as the minimum concentration that can be considered non‐zero and statistically higher than the LOB at a probability of 95%, were performed as previously described [21, 22].

A total of 34 replicates of WT‐only samples (gDNA from healthy donors), with theoretical concentrations (based on the Qubit quantifications) of 10 000 copies/PCR, were tested. The means of the numbers of false‐positive droplets were calculated for each detection. The corrected means were then calculated using the following equation: $\mu_{\text{corr}}=\mu +1.645\;\sigma \sqrt{N}$, where μ is the mean, σ is the standard deviation of false‐positive events, and N is the number of experiments performed. Notably, the LOB_95%_ were determined by fitting the $\mu_{\text{corr}}$ on Normal Law approximation and Chernoff's inequality. The theoretical LOD_95%_ were calculated following Stilla Technologies' instructions (Table S7).

### 2.9 Statistical analyses

All statistical analyses were performed using the graphpad prism software v8.0.0 (GraphPad Software, La Jolla, CA, USA). Coefficients of determination (*R* ^2^) were calculated using linear regression analyses. Coefficients of variation (CV) were calculated using the following equation: $\mathrm{CV}\;\left(\%\right)=\left(\sigma /\mu \right)\times 100$, where σ is the standard deviation and μ is the mean of the replicate results.

## 3 Results

**Fig. 1:**
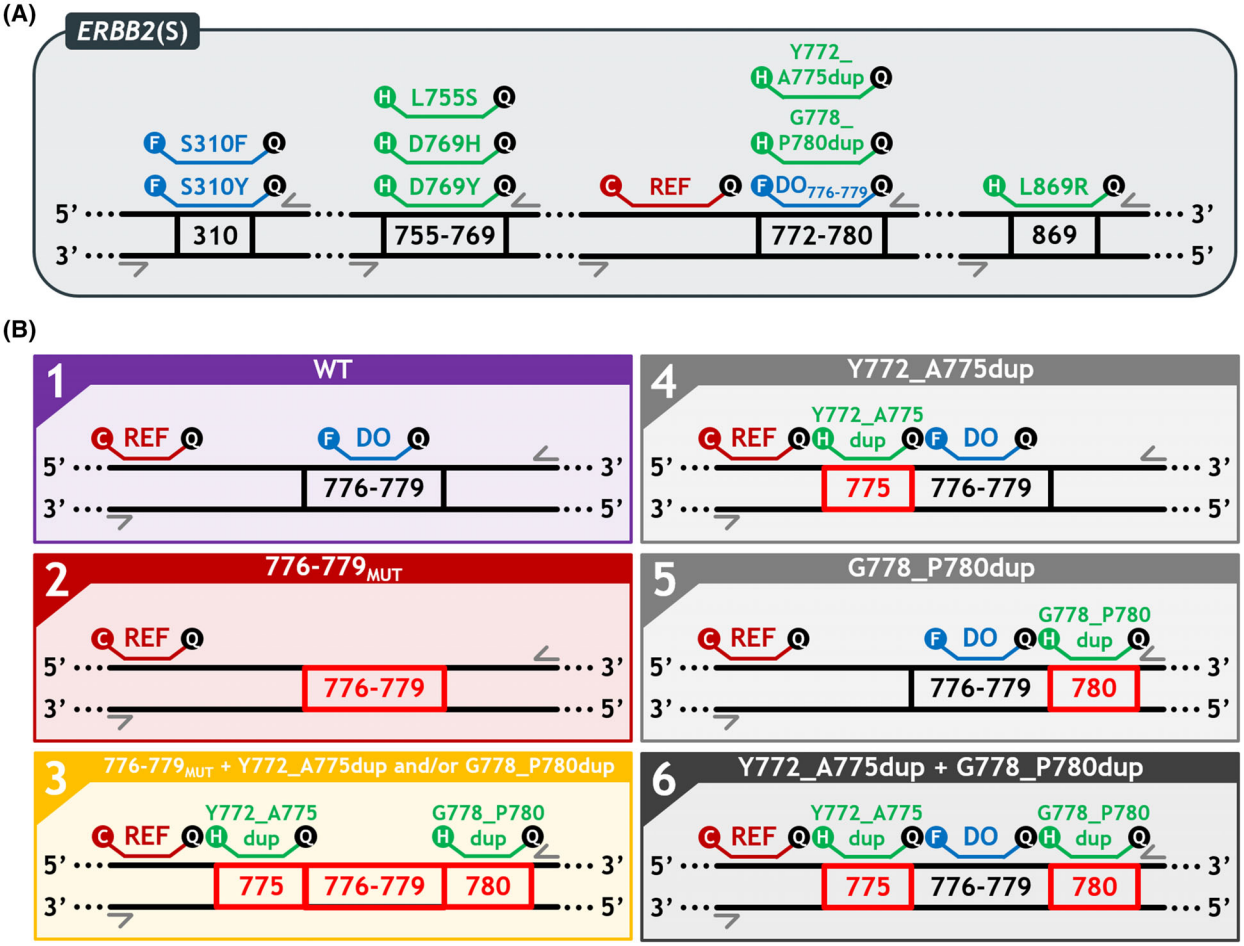
Design of the *ERBB2*(S) multiplex assay. (A) Design diagram of the *ERBB2* screening (*ERBB2*(S)) assay. Four amplicons are simultaneously amplified using four couples of primers (gray arrows) to permit the detection of the S310F and S310Y mutations with FAM‐labeled probes (blue); L755S, D769H, D769Y, Y772_A775dup, G778_P780dup, and L869R mutations with HEX‐labeled probes (green); and the mutations on codons 776–779 using a FAM‐labeled drop‐off probe (blue) combined with a Cy5‐labeled reference probe (red). (B) Representation of the different possible cases obtained with the three‐color detection strategy designed for the 772–780 amplicon. WT sequences are detected by both the drop‐off (DO) and reference (REF) probes and thus produce a FAM^+^‐Cy5^+^ double‐positive signal (case #1). Sequences mutated on codons 776–779 (776–779_MUT_) are only detected by the REF probes and produce a Cy5^+^ simple positive signal (case #2). In the case of an additional Y772_A775dup and/or G778_P780dup mutation, the signal obtained is HEX^+^‐Cy5^+^ double‐positive (case #3). Sequences with a Y772_A775dup (case #4), a G778_P780dup (case #5), or both simultaneously (case #6) produce a FAM^+^‐HEX^+^‐Cy5^+^ triple‐positive signal. C, Cy5; DO, drop‐off; F, FAM; H, HEX; MUT, mutation; REF, reference.

We designed a multiplex screening assay that can detect 17 different *ERBB2* mutations (Table S3) following the *in silico* design and verification methods previously described [21, 22]. This *ERBB2* screening assay (*ERBB2*(S) assay) (Fig. 1A) includes a drop‐off system for the detection of the 776–779 hotspot mutations (Drop‐Off_776–779_) using a FAM‐labeled drop‐off (DO) probe covering the 776–779 hotspot and a Cy5‐labeled reference (REF) probe located on the same amplicon. It has a grouped detection using two FAM‐labeled probes targeting the S310F and S310Y mutations and a grouped detection using four HEX‐labeled probes targeting the L755S, D769H, D769Y, and L869R mutations. It also has a grouped detection using two HEX‐labeled probes targeting the Y772_A775dup and G778_P780dup mutations that work in association with the DO and REF probes located on the same amplicon. WT sequences are detected by both the DO and REF probes and thus produce a FAM^+^‐Cy5^+^ double‐positive signal (Fig. 1B, case #1). Sequences mutated on codons 776–779 (776–779_MUT_) are only detected by the REF probes and produce a Cy5^+^ simple positive signal (Fig. 1B, case #2). In the case of an additional Y772_A775dup and/or G778_P780dup mutation, the signal obtained is, therefore, HEX^+^‐Cy5^+^ double‐positive (Fig. 1B, case #3). Sequences with a Y772_A775dup (Fig. 1B, case #4), a G778_P780dup (Fig. 1B, case #5), or both of them simultaneously (Fig. 1B, case #6) produce a FAM^+^‐HEX^+^‐Cy5^+^ triple‐positive signal.

### 3.1 *ERBB2* assay optimization

**Fig. 2:**
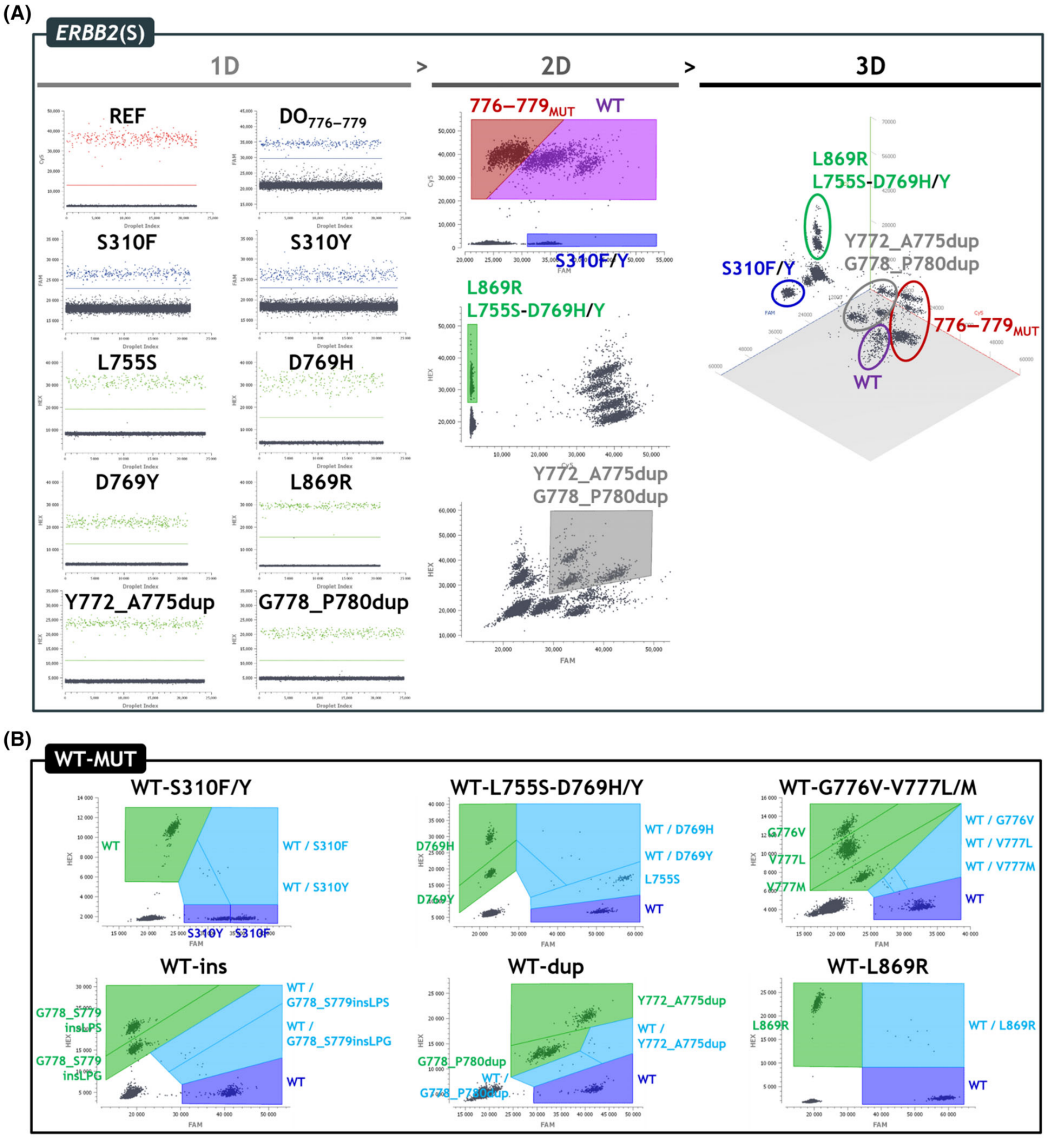
Optimization and quantification strategies for the *ERBB2* assays. (A) *ERBB2* screening (*ERBB2*(S)) assay. “1D” left panel shows optimized fluorescence signals produced by each probe in a simplex reaction. “2D” center panel shows the polygon gating quantification strategy defined with a mixture of WT gDNA and MUT gBlocks. “3D” right panel shows the cluster positions according to their relative FAM‐HEX‐Cy5 fluorescence signals. (B) Summary of the polygon gating quantification strategies defined for the six WT‐MUT duplexes. The duplexes were designed to precisely identify the mutations found with the *ERBB2*(S) assay. C, Cy5; DO, drop‐off; F, FAM; H, HEX; MUT, mutation; REF, reference.

To begin with, we have checked the quality of the signals obtained in simplex reactions (Fig. 2A, “1D” left panels) to verify the quality of the positive signals, which showed great separability from the negative signals and very low amounts of “rain” droplets (of intermediate fluorescence). We then identified the optimal oligonucleotide concentrations (Table S2), annealing/elongation temperatures, and scanning parameters (Table S5) through several optimization experiments using mixtures of WT gDNA and MUT gBlocks. For droplet classification and quantification, we used polygonal gates on the 2D dot plots (Fig. 2A, “2D” center panels) with the help of the 3D visualization for cluster identification (Fig. 2A, “3D” right panels).

Notably, we obtained three different cluster positions for the three types of duplications analyzed: Y772_A775dup (c.2313_2324dup), G778_P780dup(1) (c.2331_2339dup), and G778_P780dup(2) (c.2332_2340dup). Given the three‐color strategy designed for the detection of the Y772_A775dup and G778_P780dup mutations, the corresponding clusters appear superimposed to the WT cluster on the FAM‐Cy5 2D dot plot and are, therefore, also quantified in the “WT” gate. This phenomenon was taken into account by subtracting the number of positive droplets detected in this “WT” gate from the number of positive droplets detected in the “Y772_A775dup‐G778_P780dup” gate defined in the FAM‐HEX 2D dot plot.

We also developed six WT‐MUT duplexes to precisely identify the mutations evidenced with the *ERBB2*(S) assay (Fig. 2B).

### 3.2 *ERBB2*(S) assay validation

As described previously [21, 22], we started by determining the LOB_95%_ and LOD_95%_ for the one‐, two‐, or three‐replicate assays (Table S7). The LOB_95%_ for the one‐replicate *ERBB2*(S) assay was three droplets for the S310F/Y detection, four droplets for the L755S‐D769H/Y‐L869R detection, four droplets for the Y772_A775dup‐G778_P780dup detection, and five droplets for the 776–779_MUT_ detection. Samples with numbers of positive droplets between the LOB_95%_ and the LOD_95%_ were systematically investigated by performing two replicates (or three when needed) to increase the sensitivity.

**Fig. 3:**
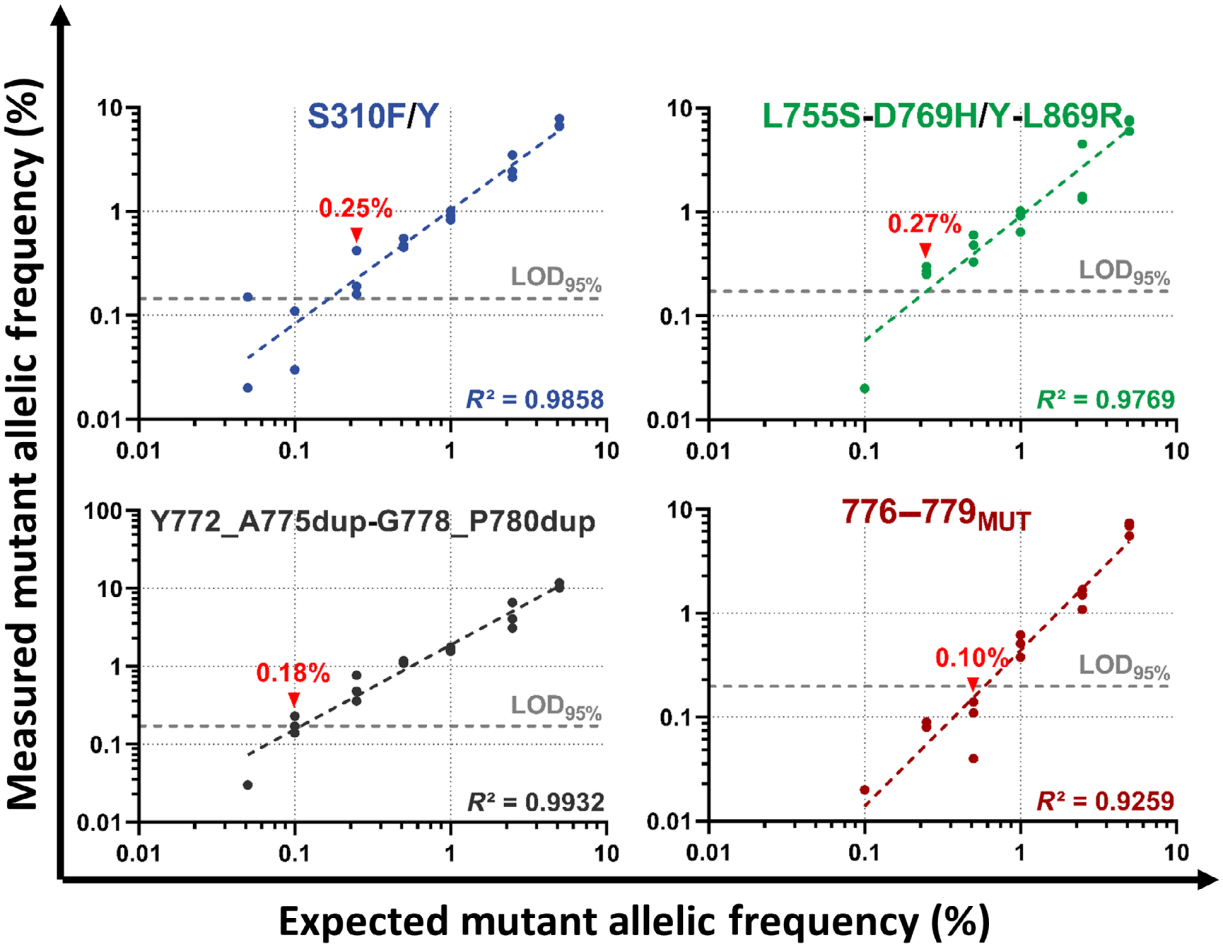
Evaluation of the sensitivity of the *ERBB2*(S) assay. DNA mixtures were prepared through serial dilutions of MUT gBlocks in a constant WT gDNA background of 10 000 copies/PCR to reach theoretical MAFs of 5%, 2.5%, 1%, 0.5%, 0.25%, 0.1%, and 0.05%. The mixtures included MUT gBlocks for S310F, S310Y, L755S, D769H, D769Y, Y772_A775dup, G778_P780dup, L869R, and the six most frequent mutations identified on codons 776–779. Any detection with at least two replicates equal to or higher than the theoretical LOD_95%_ (represented as gray, dotted lines) was considered positive. The sensitivities of each detection (displayed in red) were defined as the means of the measured MAFs (%) obtained for all replicates. MUT, mutation.

We then analyzed the sensitivity of detection using DNA mixtures prepared through serial dilutions of MUT gBlocks in a constant WT gDNA background of 10 000 copies/PCR (Fig. 3), which were assayed in triplicate (except for the dilutions at a mutant allelic frequency (MAF) = 0.05%, which were performed in quadruplicate). Any detection with at least two replicates with equal or higher numbers of positive droplets than the corresponding LOD_95%_ was considered to be positive and the means of the measured MAFs (%) for all replicates were calculated to obtain the sensitivity values. Thus, we obtained sensitivities of 0.25% for S310F/Y, 0.27% for L755S‐D769H/Y‐L869R, 0.18% for Y772_A775dup‐G778_P780dup, and 0.10% for 776–779_MUT_.

A linearity study over a dynamic range from 5 to 1000 copies/PCR (Fig. S2) was also performed, and the *R* ^2^ calculated for the linear regressions performed between expected and measured concentrations of each detection ranged from 0.9948 to 0.9999.

Finally, we studied the intra‐assay (repeatability) and inter‐assay (reproducibility) variations. The CV for the repeatability studies ranged from 3.4% to 12.5% (Table S8), and we obtained the following CV values for reproducibility: 15.9% (WT), 10.4% (S310F/Y), 9.8% (L755S‐D769H/Y‐L869R), 11.0% (Y772_A775dup‐G778_P780dup), and 11.3% (776–779_MUT_) (Fig. S3). Such CV values are expected in dPCR intra‐ and inter‐assay validation experiments for which a CV cutoff of 20% is considered acceptable, as mentioned in [24].

### 3.3 Diagnostic strategy for *ERBB2* mutation identification

**Fig. 4:**
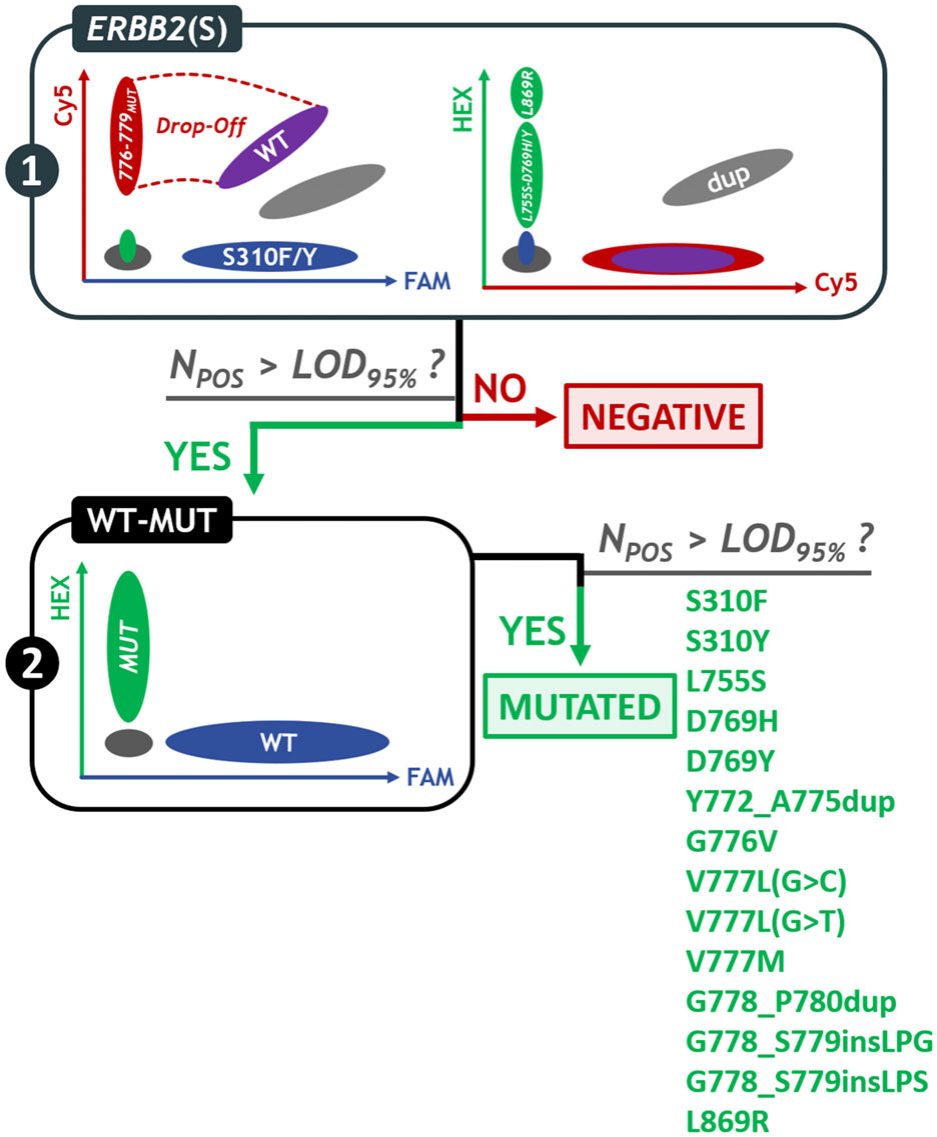
Diagnostic strategy for *ERBB2* mutation identification. The *ERBB2*(S) assay was performed as a first‐line screening assay and was followed, in case of a positive result, by the corresponding WT‐MUT Duplex assays to identify the mutation(s). C, Cy5; dup, duplications; F, FAM; H, HEX; MUT, mutation.

**Fig. 5:**
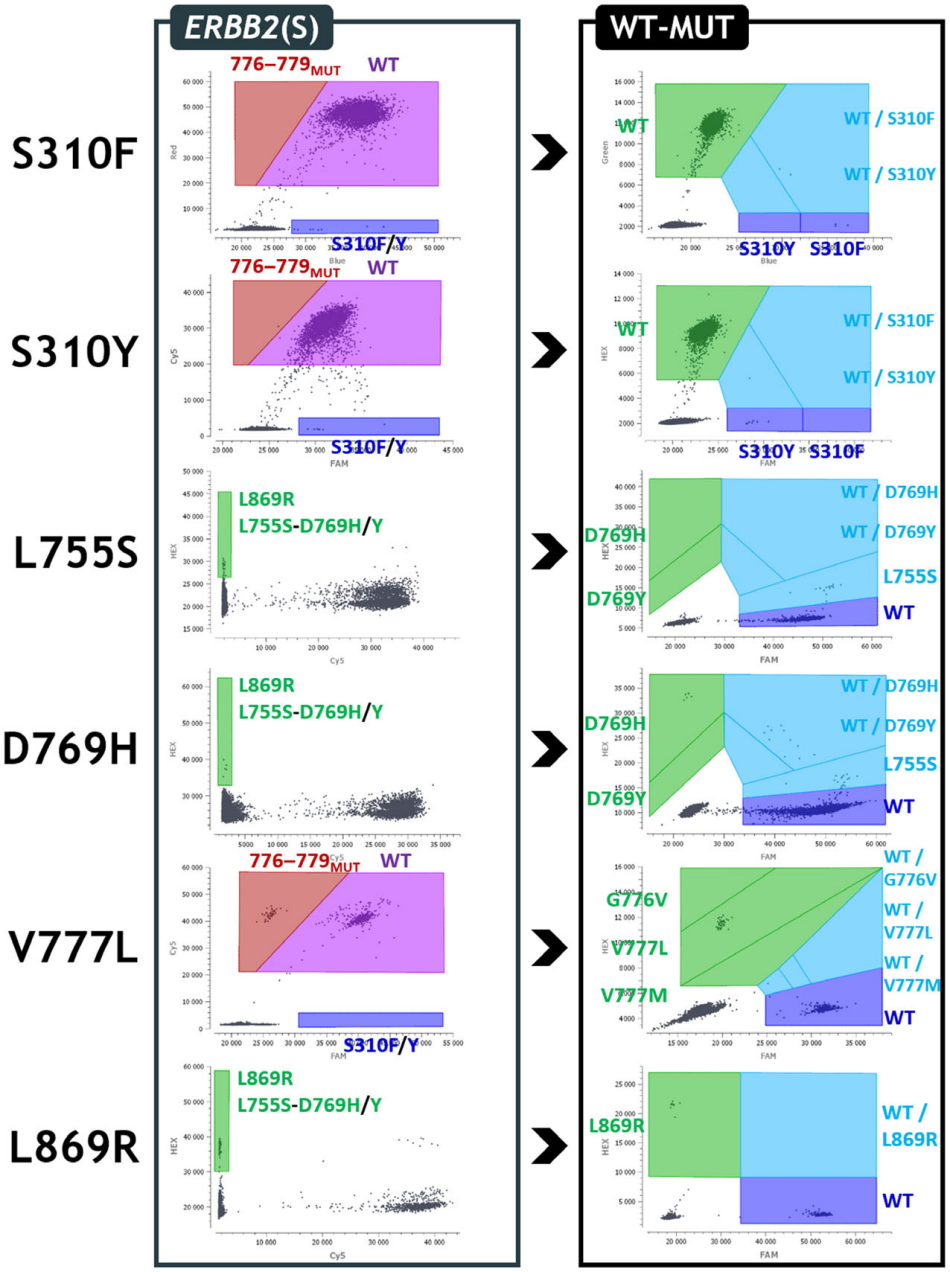
Examples of *ERBB2* mutations identified on patient cfDNA samples with the *ERBB2*(S) and WT‐MUT Duplex assays. 2D dot plot results of six *ERBB2* mutations (S310F, S310Y, L755S, D769H, V777L, and L869R). The mutations were first detected using the *ERBB2*(S) assay with the respective MAFs of 0.12%, 0.09%, 0.59%, 0.03%, 8.71%, and 7.10%, and confirmed using the corresponding WT‐MUT Duplex assay with the respective MAFs of 0.15%, 0.14%, 0.56%, 0.06%, 11.10%, and 4.02%. C, Cy5; F, FAM; H, HEX; MUT, mutation.

For the detection and identification of *ERBB2* mutations, we developed a two‐step diagnostic strategy (Fig. 4). We performed the screening assay first. If there was no evidence of *ERBB2* mutation, samples were considered negative (Fig. S4). Otherwise, we performed the WT‐MUT Duplex assays to precisely identify the mutations evidenced with the *ERBB2*(S) assay (Fig. 5).

### 3.4 *ERBB2* assay results in patients

The median cfDNA concentration for the 304 plasma samples from the 272 patients with MBC was 14.1 ng·mL^−1^ of plasma (range: 3.7–1390 ng·mL^−1^ of plasma; mean: 49.2 ng·mL^−1^ of plasma) (Dataset S1). Nine patients (3.3%) harbored at least one mutation. *ERBB2* mutation was more frequent in the ILC subtype (4.8% for mixed and pure ILC samples; 5.9% for pure ILC samples) than in the IBC‐NST subtype (2.6%). A total of 12 mutations were found, with the simultaneous detection of two mutations per sample occurring in three patients (one example is displayed in Fig. S5).

**Fig. 6:**
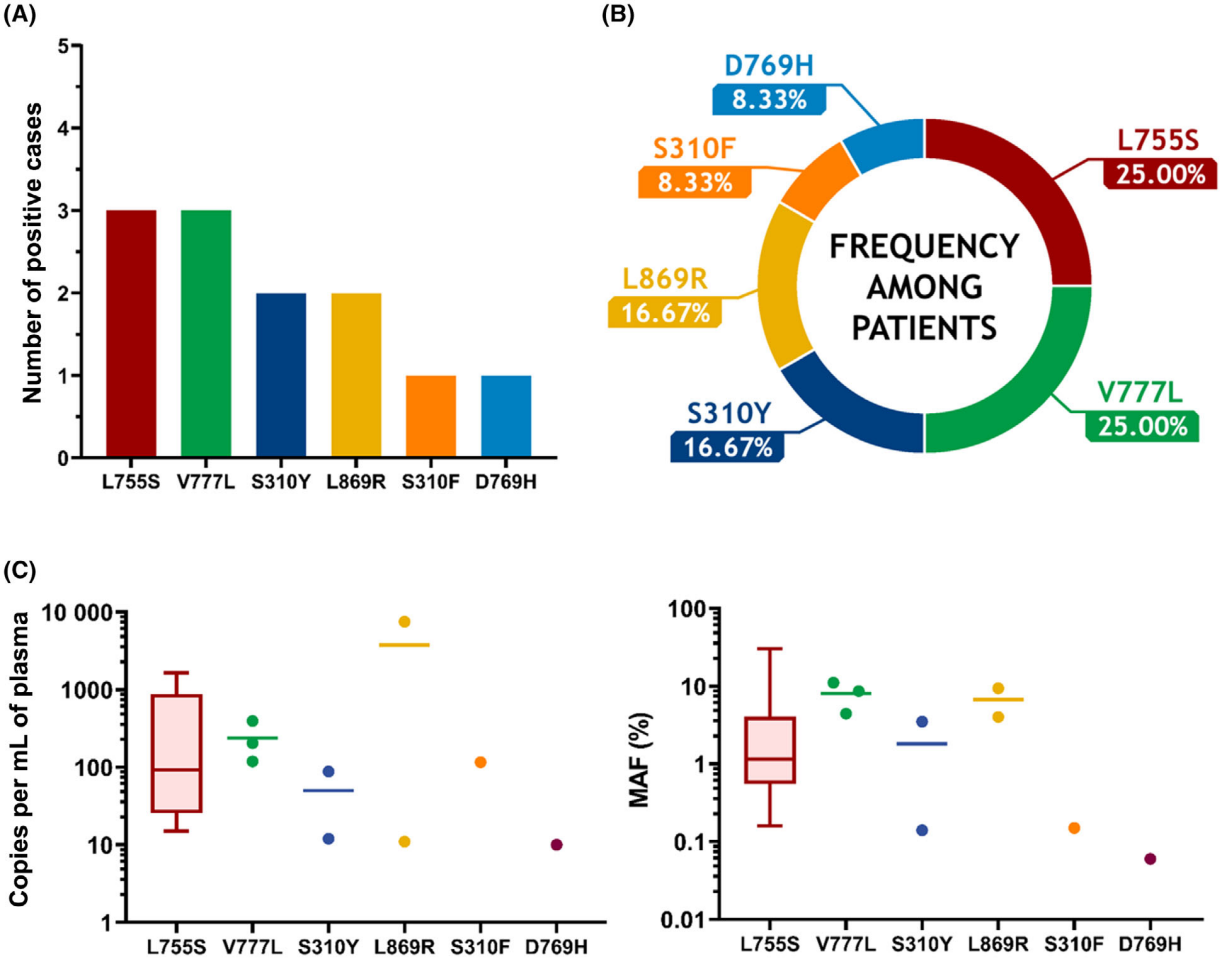
*ERBB2* mutation detection in the plasma of 272 HR+/HER2− MBC patients. (A) Number of positive cases per mutation. Results are for the *ERBB2* mutations found among the 272 HR+/HER2− MBC patients. (B) Relative frequencies of the *ERBB2* mutations identified. (C) Distributions of the mutations in copies·mL^−1^ of plasma and in MAF (%) (represented as points, with median lines for more than one instance, and as box plots for more than three instances).

Among the 17 mutations potentially detected, six were detected in our series of plasma samples with the following results: L755S (3/12, 25.00%), V777L (3/12, 25.00%), S310Y (2/12, 16.67%), L869R (2/12, 16.67%), S310F (1/12, 8.33%), and D769H (1/12, 8.33%) (Fig. 6A,B). Although few in number, the relative frequencies obtained from our series of patients were rather consistent with the frequencies listed in the COSMIC database (Table 1 and Table S9). The distribution of the quantifications obtained was quite broad in regard to copies·mL^−1^ of plasma with the following results (median (min–max)): L755S (92 (15–1648)), V777L (204 (119–395)), S310Y (50 (12–88)), L869R (3766 (11–7521)), S310F (116), and D769H (10). The distribution was similarly broad for MAFs (%), with the following values (median (min–max)): L755S (1.15 (0.16–30.19)), V777L (8.63 (4.42–11.10)), S310Y (1.82 (0.14–3.49)), L869R (6.69 (4.02–9.36)), S310F (0.15), and D769H (0.06) (Fig. 6C).

**Table 1:** *ERBB2* mutation frequencies observed in our study, the COSMIC database, and the literature. Mutation frequencies (%) are sorted from highest to lowest based on COSMIC results. A total of 437 mutations were found in three separate studies [3, 13, 17]. The protein domain impacted by each mutation and the clinical relevance reported in three studies [16, 17, 18] are also displayed. CR, complete response; ECD, extracellular domain; JMD, Juxta‐membranous domain; KD, kinase domain; NT, not tested; PR, partial response; SD, stable disease.

Mutation	Frequency reported in cosmic (%)	Frequency among 437 mutations reported elsewhere [3, 13, 17] (%)	Frequency in our study (%)	Protein domain	Clinical relevance
L755S	19.55	23.87	25.00	KD	4/8 CR, PR, or SD [16] 4/19 CR or PR [17] 5/11 SD [18]
V777L	12.17	17.82	25.00	KD	1/5 PR [16] 6/15 CR or PR [17] 3/5 SD [18]
S310F/Y	8.07	8.76	25.00	ECD	2/2 CR or SD [16] 6/10 CR or PR [17] 2/7 PR or SD [18]
D769Y/H	5.38	8.46	8.33	KD	1/1 SD [16] 1/7 CR or PR [17] 3/5 CR or SD [8]
G778_P780dup	4.80	6.34	0.00	KD	2/2 SD and PR [16] 7/8 CR or PR [17] 2/2 PR [18]
Y772_A775dup	4.23	2.11	0.00	KD	3/3 CR or SD [16] 4/7 CR or PR [17] 2/2 PR and SD [18]
L869R	2.40	2.11	16.70	KD	1/1 PR [16] 2/2 PR [18]
E770_A771insGIRD	1.54	2.72	NT	KD	
I767M	1.54	1.21	NT	KD	
R678Q	1.37	1.51	NT	JMD	
G778_S779insLPS	1.03	1.81	0.00	KD	
V842I	1.03	1.81	NT	KD	1/1 SD [18]
G778_S779insLPG	1.03	0.60	0.00	KD	
T862A	0.69	1.51	NT	KD	
D873G	0.69	0.91	NT		
G776V	0.51	1.51	0.00	KD	0/2 [16] 0/2 [17] 1/1 SD [18]
L755_T759del	0.34	1.81	NT	KD	
G727A	0.34	0.91	NT	KD	
P122L	0.00	0.60	NT	ECD	
V777_G778insGSP	0.00	0.60	NT	KD	

**Fig. 7:**
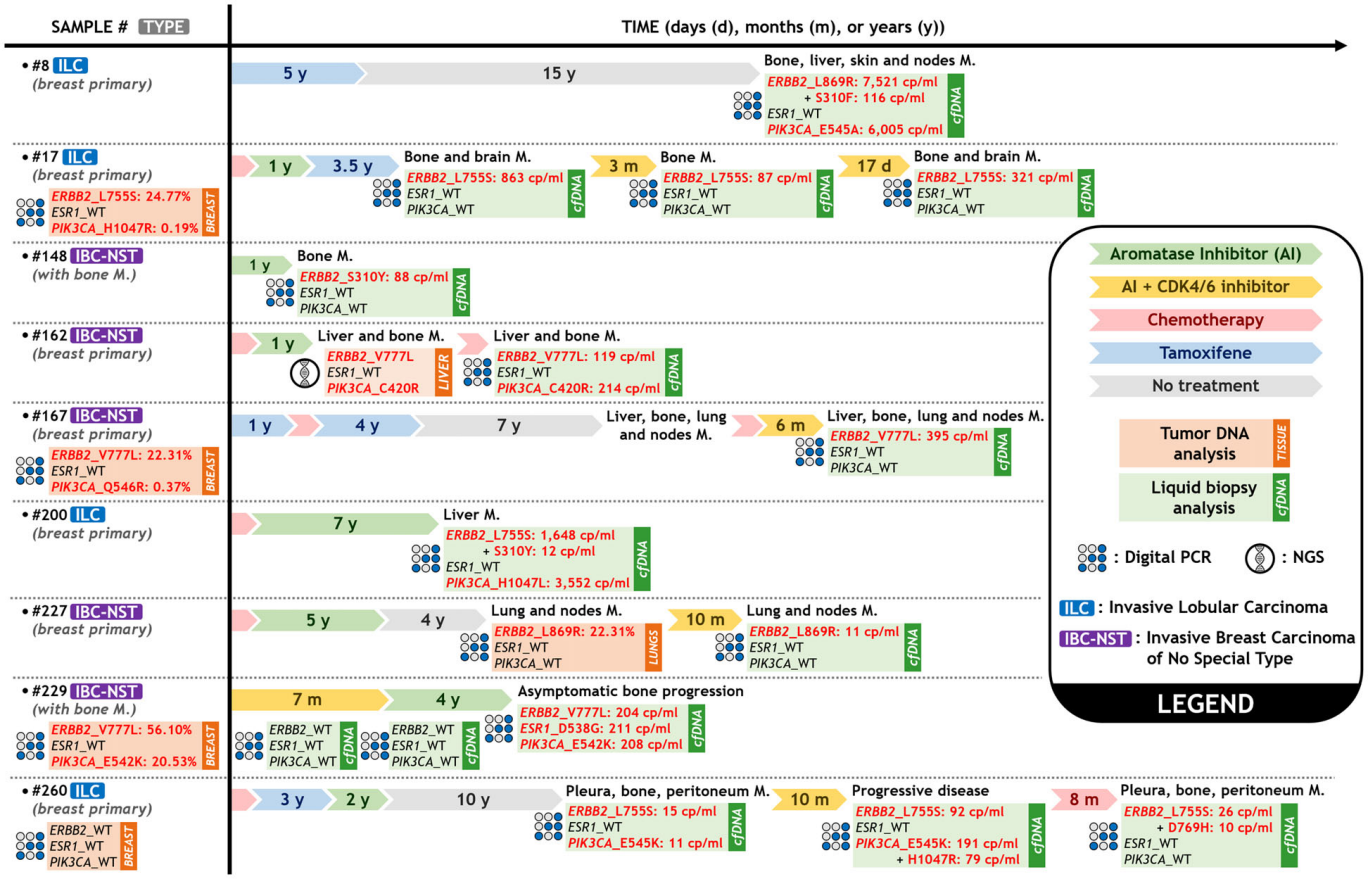
Clinical timelines for the nine patients with HR+/HER2− MBC bearing *ERBB2* mutation(s) in their cfDNA at the metastatic stage. Patient histories are shown from breast cancer diagnosis until the analysis of cfDNA. The arrows represent distinct therapies and durations (not to scale). The results of the tumor analyses are in orange squares (with tissue location). Analyses performed on cfDNA are in green squares (see legend). AI, aromatase inhibitor; M, metastases; NT, not tested; PD, progressive disease.

Five of the nine mutated patients had IBC‐NST histology, and four had ILC. Seven patients had bone metastases (three of which were bone metastases only), and four patients had liver metastases at the time of sampling for cfDNA analysis. The treatments patients received prior to *ERBB2* mutation testing by liquid biopsy are shown in Fig. 7. For patients #8, #162, #200, #229, and #260, a phosphatidylInositol‐4,5‐bisphosphate 3‐kinase catalytic subunit alpha (*PIK3CA*) mutation was also found at relatively comparable concentrations, suggesting the presence of a tumor clone expressing both types of mutation. In only one case (patient #229), we observed the emergence of an *ESR1* mutation in addition to *ERBB2* and *PIK3CA* mutations (Fig. 7).

For two patients, we performed a longitudinal follow‐up of *ERBB2* mutation levels. Patient #17 was tested for the onset of bone metastases after 4.5 years of hormone therapy. Treatment with cyclin‐dependent kinase 4/6 inhibitor and aromatase inhibitor was initiated. Three months after the beginning of treatment, the mutation concentration had fallen from 863 to 87 copies·mL^−1^. At the same time, brain metastases were discovered in this patient; and an increase in the mutation rate was observed about 10 days later. Ten years after the end of adjuvant ET, pleura, bone, and peritoneum metastases were discovered for patient #260. *ERBB2* and *PIK3CA* mutations were found at low concentrations (15 and 11 copies·mL^−1^, respectively). After a favorable clinical and biological course under CDK4/6 inhibitor and aromatase inhibitor treatment, disease progression was accompanied by an increase in mutation concentrations and the emergence of a new *PIK3CA* mutation (H1047R). A few months of chemotherapy showed efficacy on bone metastases. Unfortunately, there was a new progression with the appearance of liver metastases accompanied by a decrease in the concentration of the L755S mutation and the emergence of the D769H mutation (Fig. 7).

Six matched tumors (four initial breast tumors and two metastatic samples) were available in patients with an *ERBB2* mutation identified in plasma. One patient (#162) was tested by next‐generation sequencing (NGS) in the frame of the SAFIR02 clinical trial (NCT02299999) (Fig. 7). The median percentage of tumor cells for the samples was 60% (range: 40–80%). The median time between tumor and first plasma collections was 4 years (range: 0.3–15 years). For five of these patients, the *ERBB2* mutation identified in plasma could also be detected in tumor tissues (L755S for patient #17, V777L for patient #162, V777L for patient #167, L869R for patient #227, and V777L for patient #229). No mutation was revealed in tumor tissue for patient #260, but an L755S mutation followed by an L755S‐D769H double mutation was identified in plasma (Fig. S6). For this patient, the initial tumor tested had the longest time between tumor and first plasma collections (15 years) compared to the five other patients (4.4, 0.3, 13.5, 0.9, and 3.7 years, respectively). Thus, the concordance rate of *ERBB2* mutation status between plasma cfDNA and tumor tissue was 83% (5/6).

## 4 Discussion

We have developed multiplex assays on the three‐color Crystal dPCR™ naica® platform (Stilla Technologies) to detect *ERBB2* mutations in the plasma. Our screening test detects 71% of all pathogenic *ERRB2* mutations described for breast carcinoma tumor samples in the COSMIC database. The assay covers all mutations representing a frequency > 2%. It has a coverage rate of 99.2% for the 776–779 hotspot mutations that appear to predict response to neratinib [18]. In cases of a positive result with the screening test, we performed a second test to identify the mutation(s).

This dPCR assay was applied to our population of 272 patients with HR+/HER2− MBC. The assay found that 3.3% (9/272) patients had one or more *ERBB2* mutations in their ctDNA. This proportion is comparable to Bertucci et al. [5] with whole‐exome sequencing of 381 HR+/HER2−metastatic samples; they found *ERBB2* activating mutations in 3% of patients. In our series of patients, *ERBB2* mutation was more frequent in the ILC subtype (5.9% for pure ILC samples), in accordance with the literature. Desmedt et al. reported *ERBB2* mutation in 5.1% of 413 primary pure ILC samples analyzed by targeted sequencing (4.3% for the 371 ER+/HER2− patients). Other studies involving smaller numbers of patients also reported positivity rates between 4% and 5% for primary ILC tumors [11, 12, 13].

The most frequent mutations in this study were L755S, V777L, S310F/Y, and D769Y/H, which is in accordance with the literature and the COSMIC database. All these mutations are located in the *ERBB2* tyrosine kinase domain, with the exception of S310F/Y mutations which are located in the *ERBB2* extracellular domain. Clinical efficacy has been demonstrated in some patients carrying these mutations and treated with neratinib (Table 1) [17, 18, 19].

We did not identify any *ERBB2* exon 20 insertion/duplication mutation. This type of alteration is most frequently reported in lung cancer [25]. G778_P780dup and Y772_A775dup, both detectable by our test, account for eight (5%) of 142 mutations detected in 5605 cases of recurrent and MBC [4] and 10 (9%) of the mutations detected in 81 patients with *ERBB2* mutant MBC [18]. On the other hand, these mutations account for < 1% of the 108 mutations detected in 2534 patients with primary or MBC in Kalra et al. [14] and 2% of the 43 HR+/HER2− MBC harboring *ERBB2* mutations in another study [9]. Some patients with this type of mutation have shown clinical responses to HER2 kinase inhibition, so it is important to identify them. It should be noted that the standard nomenclature [26] is not always applied to these variants, which can be a source of confusion. For example, Y772_A775dup and G778_P780dup (recommended names for duplication variants) can be found as A775‐G776insYVMA and P780_Y781insGSP, respectively.

In a third of the nine positive patients (3/9), two *ERBB2* mutations were simultaneously identified, which was probably made possible by the high sensitivity of our assays, allowing detection of additional mutations with MAF as low as 0.06%. In two cases, the allelic frequencies were very different for the two mutations, indicating sub‐clonality. It should be noted that a high proportion of patients acquire new *ERBB2* mutations with disease progression after receiving clinical benefits on neratinib [18]. None of the three patients in our cohort received treatment targeting the HER2 pathway.

Clinical follow‐up with repeated analysis over time was possible for 22 patients (with up to eight analysis points for one patient) and led to the detection of two mutations (patient #229, sample #8: V777L (11.1%), and patient #260, sample #3: D769H (0.06%)). This underlines the importance of monitoring assays for the analysis of liquid biopsies, for which the dPCR approach is well suited.

We could have expected higher positive ILC samples at the metastatic stage: Kalra et al. [14] found as high as 10.8% of samples with *ERBB2* mutations. This difference can be explained, in part, by the difference in technological approaches. Our dPCR assay, as well as some targeted sequencing, does not detect very rare mutations. In breast cancer, *ERBB2* mutations occur most frequently in exons encoding the active site of the tyrosine kinase domain, particularly exons 19 and 20 (56% of all mutations in Robichaux et al.) [25]. However, the spectrum of mutations in the *ERBB2* gene is broad, with many mutations described in only one sample (named “private mutations”). In the Kalra et al. study, 38 different mutations were reported, 21 of which were private. These mutations represented 20% of the 108 positive cases [14]. Whether it is important or not to look for these mutations remains unclear. It seems difficult to determine if these mutations are pathogenic with *in vivo* tests. It may also be challenging to correlate response to treatment with the presence of minor mutations within a clinical trial. A clue might be provided in the amended SUMMIT trial (NCT01953926); 14/15 patients with mutations not known to be hotspot did not respond to neratinib [18]. Without further data, identifying these ultra‐minority mutations does not appear to be essential, although they may be of particular clinical interest in the future and maybe more appropriately identified using a more exhaustive NGS approach, as presented in Jhaveri et al. [27].

One of the hypotheses underlying the higher frequency of *ERBB2* mutations at the metastatic stage is that they appear under the selective pressure of hormone therapy. Nayar et al. [6] found no mutations in six of eight primary tumors (75%) when comparing the tumors to eight paired metastatic samples harboring *ERBB2* mutation. We tested the initial tumor in four patients with mutations at the metastatic stage of the disease. In only one case, the mutation was not present initially. The “new” *ERBB2* mutation was unlikely to have arisen because of hormone therapy, as the patient had not been treated during the 10 years prior to sampling. Our results, although based on a few samples, do not support the hypothesis of *ERBB2* mutation being acquired because of ET resistance. In the three cases of the initial tumor with an *ERBB2* mutation, the mutations were present with a high allelic frequency. Concomitant *PIK3CA* mutations were also observed with a much lower allelic frequency in two cases. Interestingly, for these latter cases, only *ERBB2* mutations were found in the ctDNA at progression, thus proving the driver character of this mutation.

However, these assumptions must be weighed against the relatively small sample size of this study, which would require a larger validation cohort to confirm these results, given the low frequency of *ERBB2* mutation detection. It would also be interesting to compare these results with matched tumor samples NGS analyses for patients found negative for *ERBB2* mutation on cfDNA samples (which has not been done for economic reasons). What could also improve the specificity of these results would be to perform analyses on matched normal DNA for “suspicious” mutant allelic frequency results, as some *ERBB2* mutations have been reported to be linked with clonal hematopoiesis [28]. Although neratinib in combination with trastuzumab and fulvestrant showed promising results, it is not FDA‐approved at this time. However, as the drug development in the field of targeted therapies seems to be moving toward mutation‐specific agents, we could expect a large‐scale clinical utility of this type of assay in the near future, not only for breast cancer but also for other solid tumors. Studies have recently shown, for example, that *ERBB2*‐mutant cancers are sensitive to antibody–drug conjugates (trastuzumab deruxtecan) [29, 30].

## 5 Conclusions

Molecular analysis of long‐stored tissue is not always possible, and mutations can emerge during progression. Thus, cfDNA analysis for *ERBB2* mutation detection is a good option. Therapeutic possibilities are still limited, but several clinical and pre‐clinical studies are underway to develop and test more selective HER2 inhibitors, HER2‐inhibiting antibodies, or combinations of these drugs. Therefore, the search for *ERBB2* mutations will become part of the standard management of breast and other cancers, as *ERBB2* mutations are found across multiple cancers (e.g., uterine, cervical, colorectal, and lung) [16]. Although the dPCR approach is more restrictive in terms of the spectrum of targeted mutations, it is now widely accepted that this technology is complementary to NGS panel‐based approaches that can capture a broader spectrum of variants, especially for liquid biopsy samples for which the sensitivity of detection is crucial. Indeed, given its relatively low cost and ease of use, dPCR allows repeated sampling for longitudinal follow‐up of patients, which is of particular interest here because (a) *ERBB2* mutations can occur under the selective pressure of treatment, and (b) metastasis is a continuous and dynamic process with the possible emergence of several heterogeneous metastatic lesions; sequential testing with ctDNA analysis allows to detect these molecular alterations and to adapt treatment, when possible. Given the current state of the literature [18] the analysis can be limited in a first line to hotspot mutations that can easily be targeted with dPCR multiplex assays. Therefore, due to its high sensitivity and robustness, dPCR is well suited to the clinical detection of *ERBB2* mutations at a lower cost than NGS approaches [7].

## Conflict of interest

An “ICMJE Conflict of Interest form” was provided by each author. The following is a list of conflicts of interest according to the forms. Grants or contracts from any entity: TDMR (MSD, Novartis, Pfizer, Seagen); Consulting fees: VD (Roche Genentech, Lilly, Daiichi Sankyo, AstraZeneca, Seagen, Pierre Fabre Oncologie, Menarini, Pfizer, Novartis, Gilead, MSD, Eisai, Medac) and TDMR (AstraZeneca, GSK, Clovis Oncology, Pfizer, Gilead Sciences, Seagen, Sanofi); Payment or honoraria for lectures, presentations, speakers bureaus, manuscript writing, or educational events: CL‐P (Pfizer), VD (Pfizer, Daiichi Sankyo, AstraZeneca, Seagen, Lilly, Gilead Sciences, MSD, Roche Genentech), and TDMR (GSK, MSD); Support for attending meetings and/or travel: LC (Janssen, Ipsen); CL‐P (Novartis, GLK, Pfizer), VD (Roche Genentech, Lilly, Daiichi Sankyo, MSD, Seagen, Pfizer, Novartis, AstraZeneca, Gilead), and TDMR (Pfizer, Gilead Sciences, Eisai); Participation on a Data Safety Monitoring Board or Advisory Board: CL‐P (Pfizer, Roche, Daiichi Sankyo), VD (Roche Genentech, Daiichi Sankyo, AstraZeneca, Sanofi Aventis), and TDMR (MSD).

## Author contributions

TDMR and VQ contributed to the conception and design of the study. AC, FLD, LR, HB, AB, LC, CP, CL‐P, VD, and TDMR provided the biological material and clinical data from patients. JC designed the dPCR assays. JC, VQ, FG, and MC analyzed the genomic results. JC and VQ drawn the figure images. All authors interpreted the data and drafted, revised, and approved the manuscript.

### Peer review

The peer review history for this article is available at https://www.webofscience.com/api/gateway/wos/peer‐review/10.1002/1878‐0261.13592.

## Supporting information


**Fig. S1.** Mutated gBlocks selection for the 776–779_MUT_ detection of the *ERBB2*(S) assay and relative positions of the MUT clusters obtained with the Drop‐Off_776‐779_ system.
**Fig. S2.** Evaluation of the linearity of the *ERBB2*(S) assay.
**Fig. S3.** Evaluation of the reproducibility of the *ERBB2*(S) assay.
**Fig. S4.** Examples of negative results obtained on cfDNA samples with the *ERBB2*(S) assay and WT‐MUT duplexes.
**Fig. S5.** Example of the simultaneous detection of two mutations in a cfDNA sample with the *ERBB2* assays.
**Fig. S6.** Results of the *ERBB2*(S) and WT‐MUT Duplex assays performed on patient tumor tissue samples.
**Table S1.** Completed dMIQE2020 checklist.
**Table S2.** Oligonucleotides composing the *ERBB2* assays.
**Table S3.** Mutations detected by the *ERBB2*(S) assay.
**Table S4.** Mutated gBlocks used in this study.
**Table S5.** PCR program used for the *ERBB2* assays.
**Table S6.** Scanning parameters applied for the *ERBB2* assays.
**Table S7.** LOB_95%_ and theoretical LOD_95%_ values for the *ERBB2*(S) assay.
**Table S8.** Results obtained during the repeatability study for the *ERBB2*(S) assay.
**Table S9.** Comparison of mutation frequencies observed in the COSMIC database and our series of plasma samples.

**Dataset S1.** Results of *ERBB2* mutation detection on cfDNA samples and matched tumor samples obtained from our cohort of 272 patients with HR+/HER2‐ metastatic breast cancer.

## Data Availability

The data generated during the current study are available from the corresponding author upon request.
