# Supplementary material for: Plasma‐based analysis of ERBB2 mutational status by multiplex digital PCR in a large series of patients with metastatic breast cancer
Source: Mol Oncol. 2024 Jan 29;18(11):2714–29. doi: 10.1002/1878-0261.13592 (PMC11547241; doi:10.1002/1878-0261.13592)
Supplement: Supplementary file 1 — Fig. S1. Mutated gBlocks selection for the 776–779MUT detection of the ERBB2(S) assay and relative positions of the MUT clusters obtained with the Drop‐Off776‐779 system. Fig. S2. Evaluation of the linearity of the ERBB2(S) assay. Fig. S3. Evaluation of the reproducibility of the ERBB2(S) assay. Fig. S4. Examples of negative results obtained on cfDNA samples with the ERBB2(S) assay and WT‐MUT duplexes. Fig. S5. Example of the simultaneous detection of two mutations in a cfDNA sample with the ERBB2 assays. Fig. S6. Results of the ERBB2(S) and WT‐MUT Duplex assays performed on patient tumor tissue samples. Table S1. Completed dMIQE2020 checklist. Table S2. Oligonucleotides composing the ERBB2 assays. Table S3. Mutations detected by the ERBB2(S) assay. Table S4. Mutated gBlocks used in this study. Table S5. PCR program used for the ERBB2 assays. Table S6. Scanning parameters applied for the ERBB2 assays. Table S7. LOB95% and theoretical LOD95% values for the ERBB2(S) assay. Table S8. Results obtained during the repeatability study for the ERBB2(S) assay. Table S9. Comparison of mutation frequencies observed in the COSMIC database and our series of plasma samples. [file MOL2-18-2714-s002.docx]

**
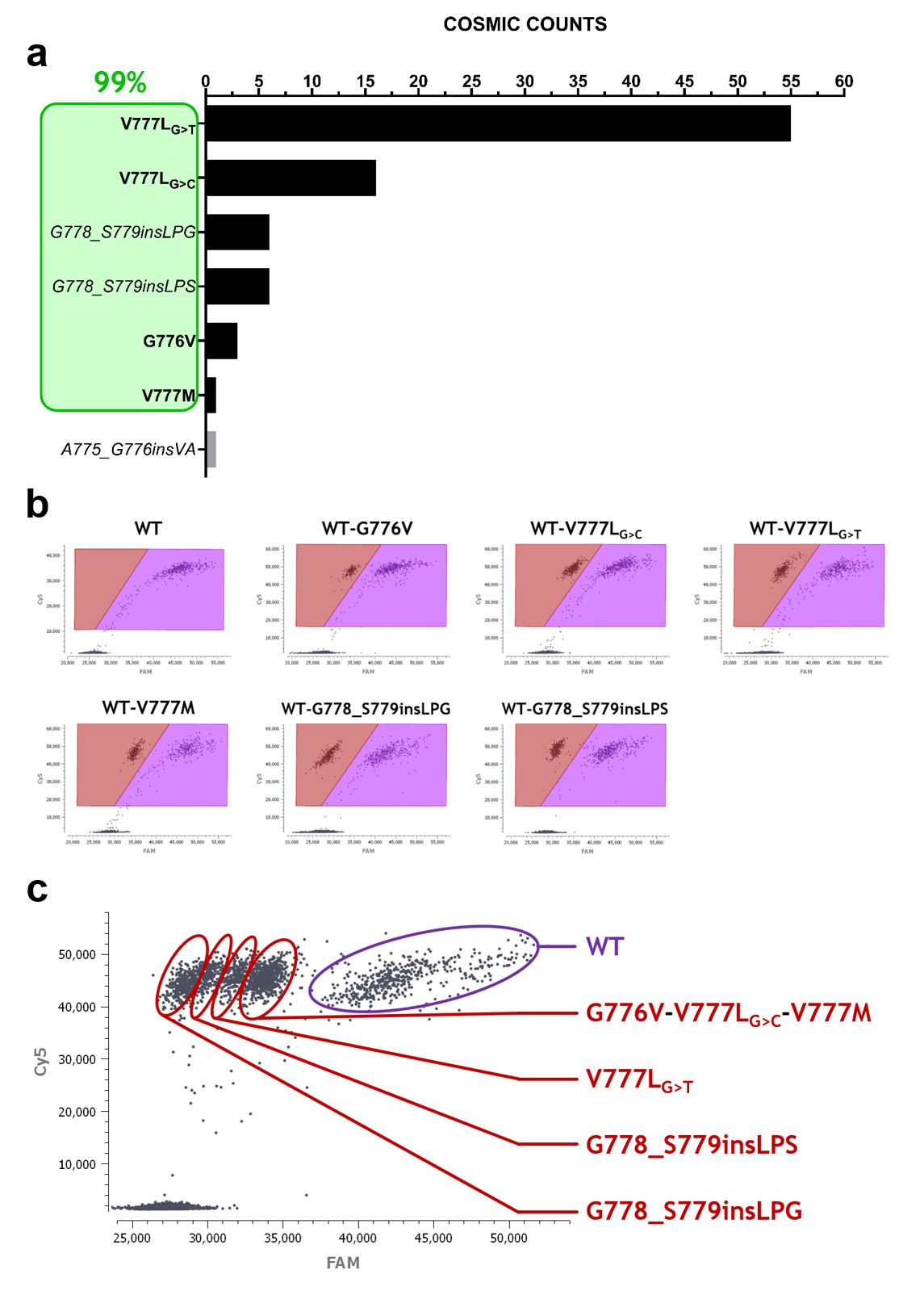
**

**Supplementary Figure S1. Mutated gBlocks selection for the 776-779_MUT_ detection of the *ERBB2*(S) assay and relative positions of the MUT clusters obtained with the Drop-Off_776-779_ system.** (**a**) Four pathogenic and three non-pathogenic (*italic*) *ERBB2* mutations on codons 776-779 were identified in the COSMIC database for breast carcinoma tumor samples. Mutations were classified based on their relative COSMIC counts; the six most frequent mutations were selected, representing 99% of all *ERBB2* mutations occurring on codons 776-779. (**b**) 2D dot plot results show the individual cluster positions obtained with the Drop-Off_776-779_ system for the selected MUT gBlocks. (**c**) 2D dot plot results show all MUT cluster positions obtained for the selected MUT gBlocks with the Drop-Off_776-779_ system, creating a useful cartography for mutation identification based on cluster position.

**
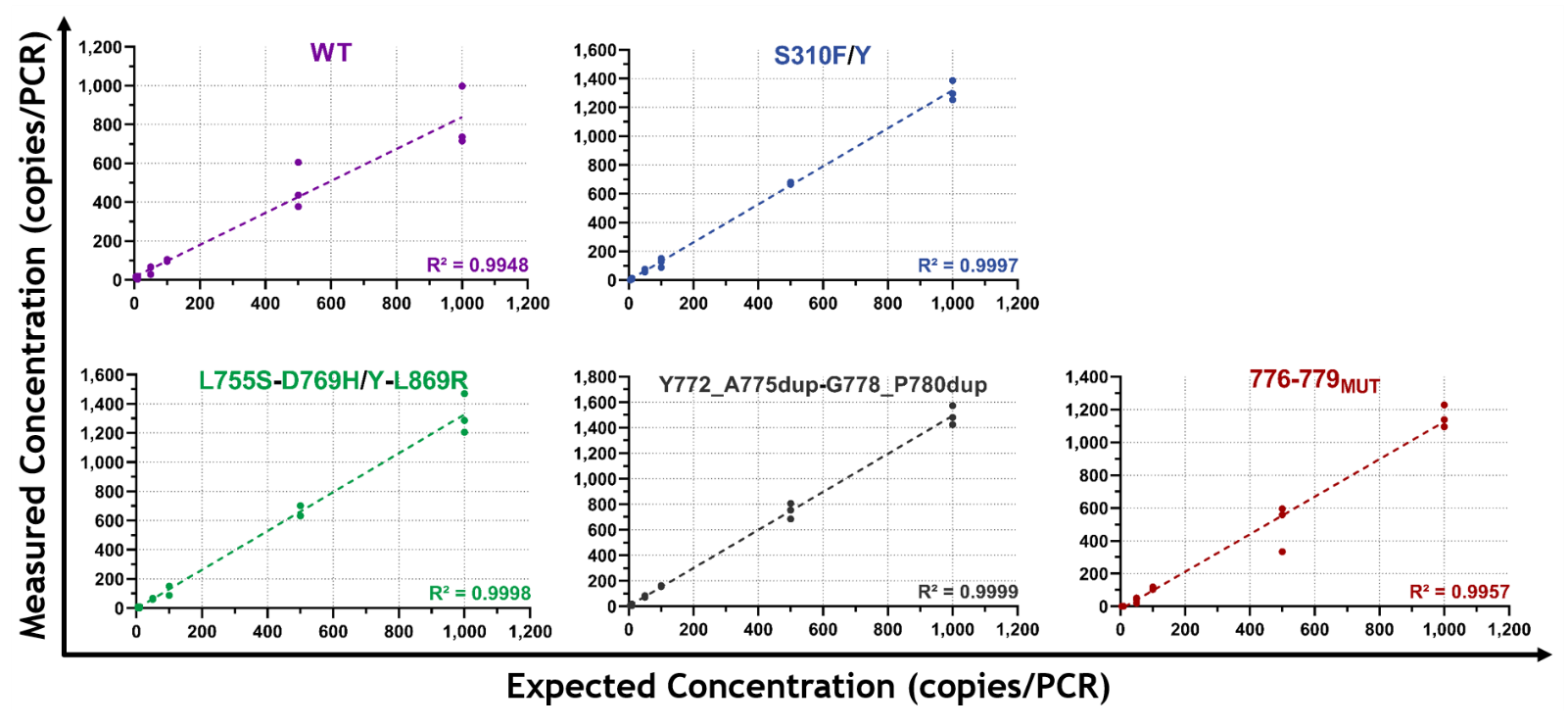
**

**Supplementary Figure S2. Evaluation of the linearity of the *ERBB2*(S) assay.** Six DNA mixtures were prepared for each multiplex assay. Preparation included serial dilutions of MUT gBlocks and WT gDNA to obtain mixtures with theoretical concentrations of 5, 10, 50, 100, 500, and 1,000 copies/PCR for each detection. Dilutions were all assayed in triplicate. A mix containing the six most frequent mutations identified on codons 776-779 (G776V, V777L_G>C_, V777L_G>T_, V777M, G778_S779insLPG, and G778_S779insPLS) was prepared to reach the same theoretical concentrations of the total amounts of MUT DNA per detection.

**
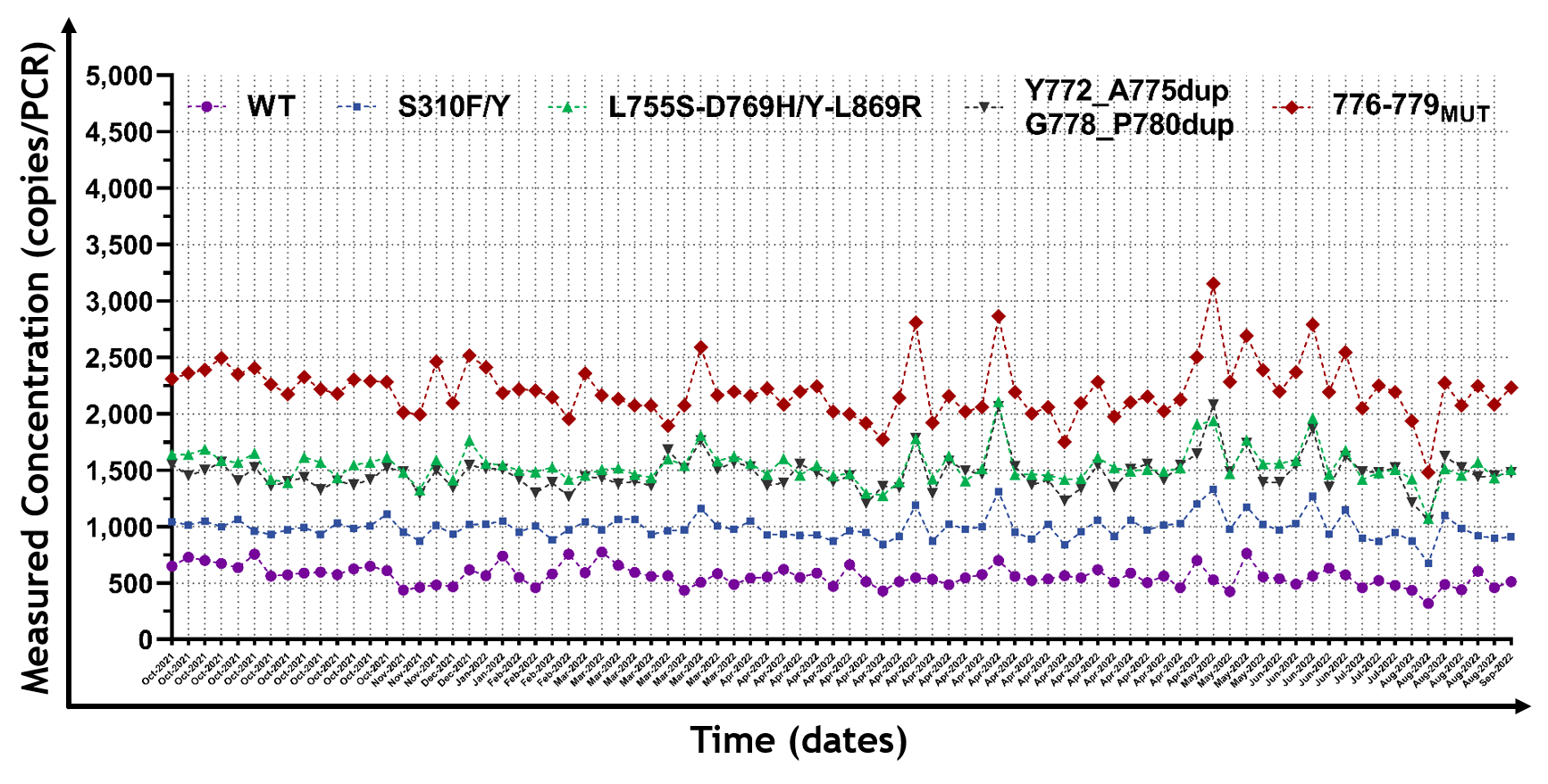
**

**Supplementary Figure S3. Evaluation of the reproducibility of the *ERBB2*(S) assay.** A DNA mix was prepared by assembling the corresponding MUT gBlocks and WT gDNA. This mixture was stored at -20°C in individual aliquots to serve as positive controls for each experiment. A total of 82 experiments were performed. The coefficients of variation (CV) for the detections were 15.9% (WT), 10.4% (S310F/Y), 9.8% (L755S-D769H/Y-L869R), 11.0% (Y772_A775dup-G778_P780dup), and 11.3% (776-779_MUT_).

**
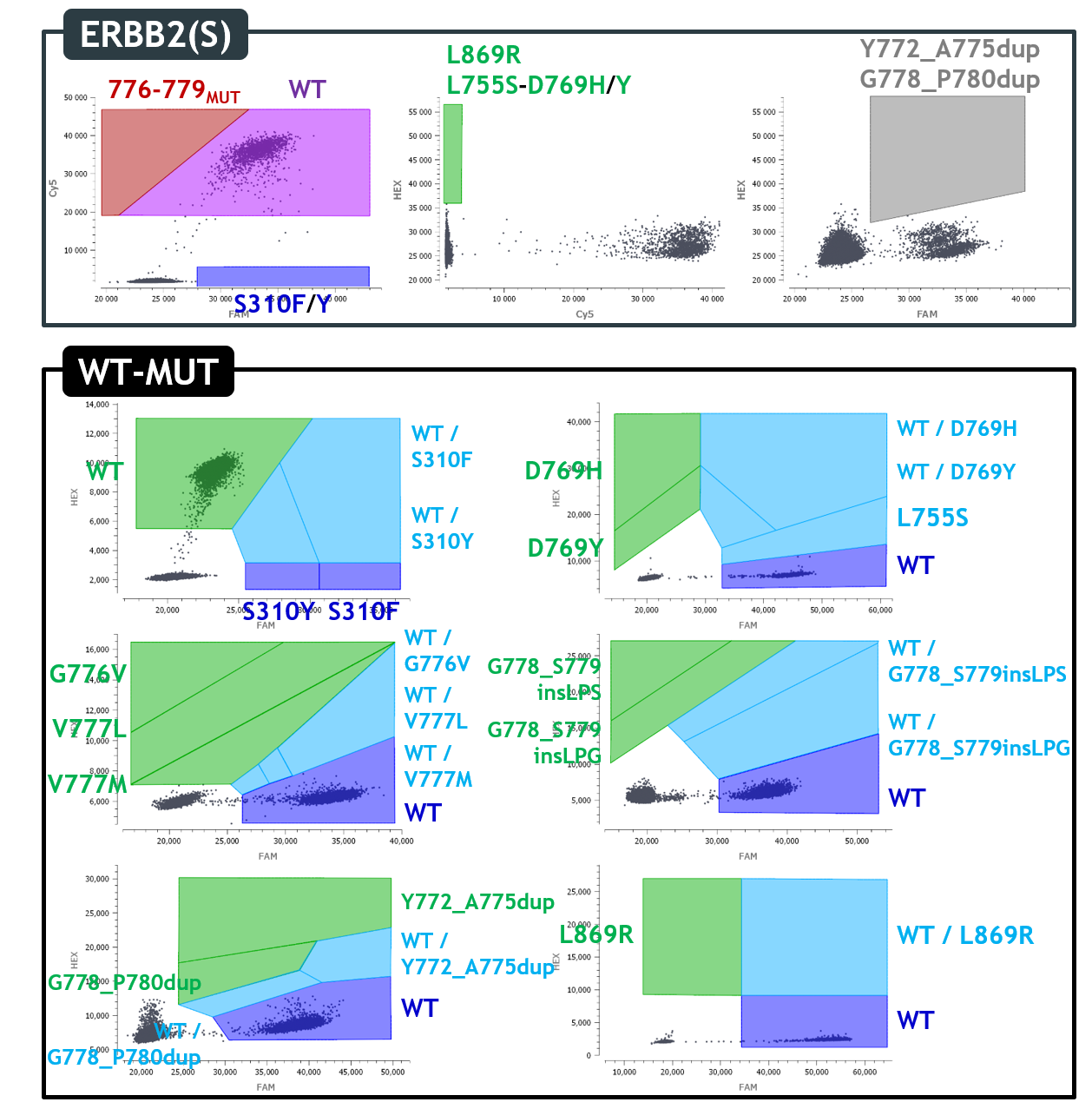
**

**Supplementary Figure S4. Examples of negative results obtained on cfDNA samples with the *ERBB2*(S) assay and WT-MUT duplexes.** 2D dot plot results show examples of WT results obtained with the *ERBB2*(S) assay and the six WT-MUT duplexes.

**
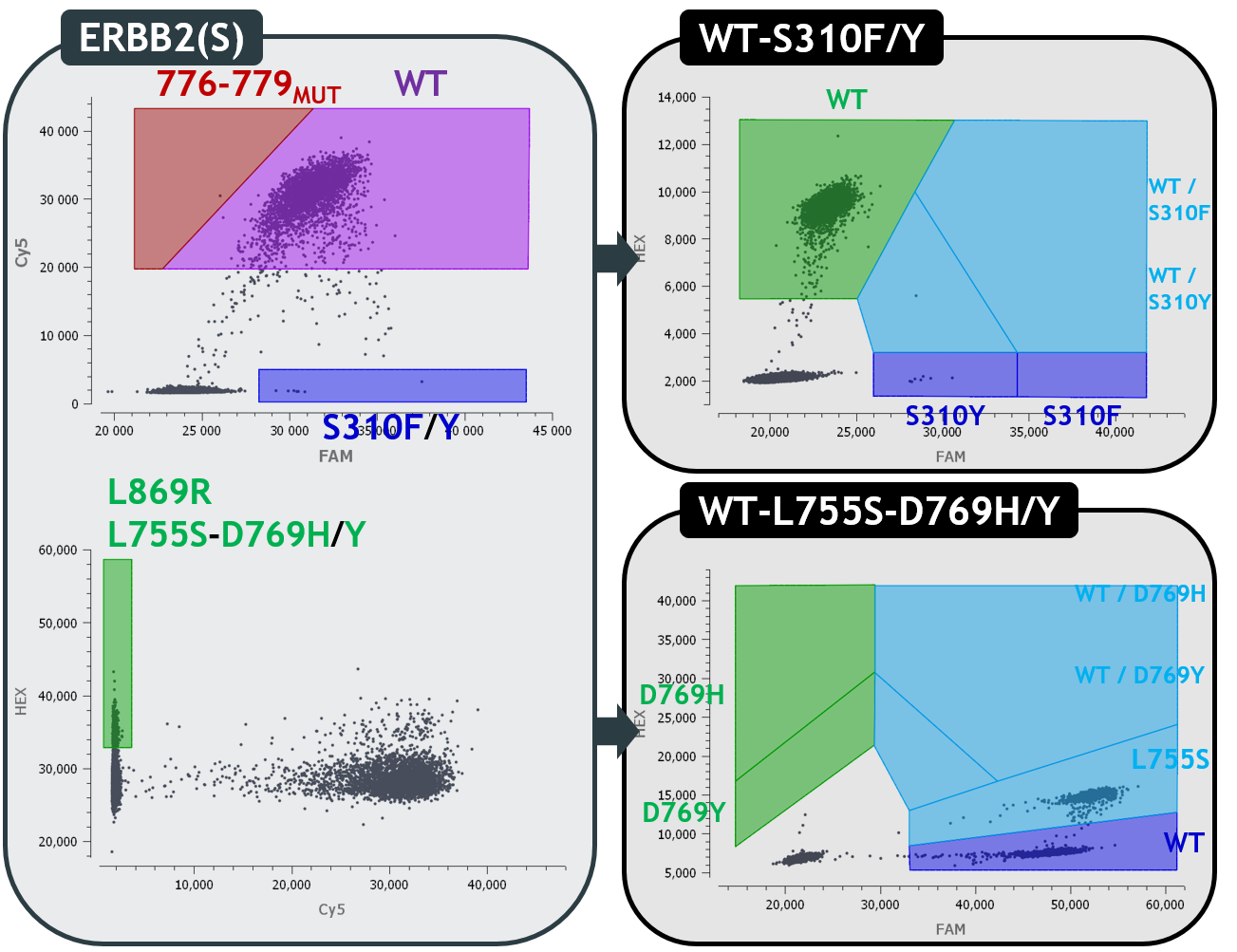
**

**Supplementary Figure S5. Example of the simultaneous detection of two mutations in a cfDNA sample with the *ERBB2* assays.** 2D dot plot results show an example of an S310Y-L755S double-mutation obtained with the *ERBB2* assays.

**
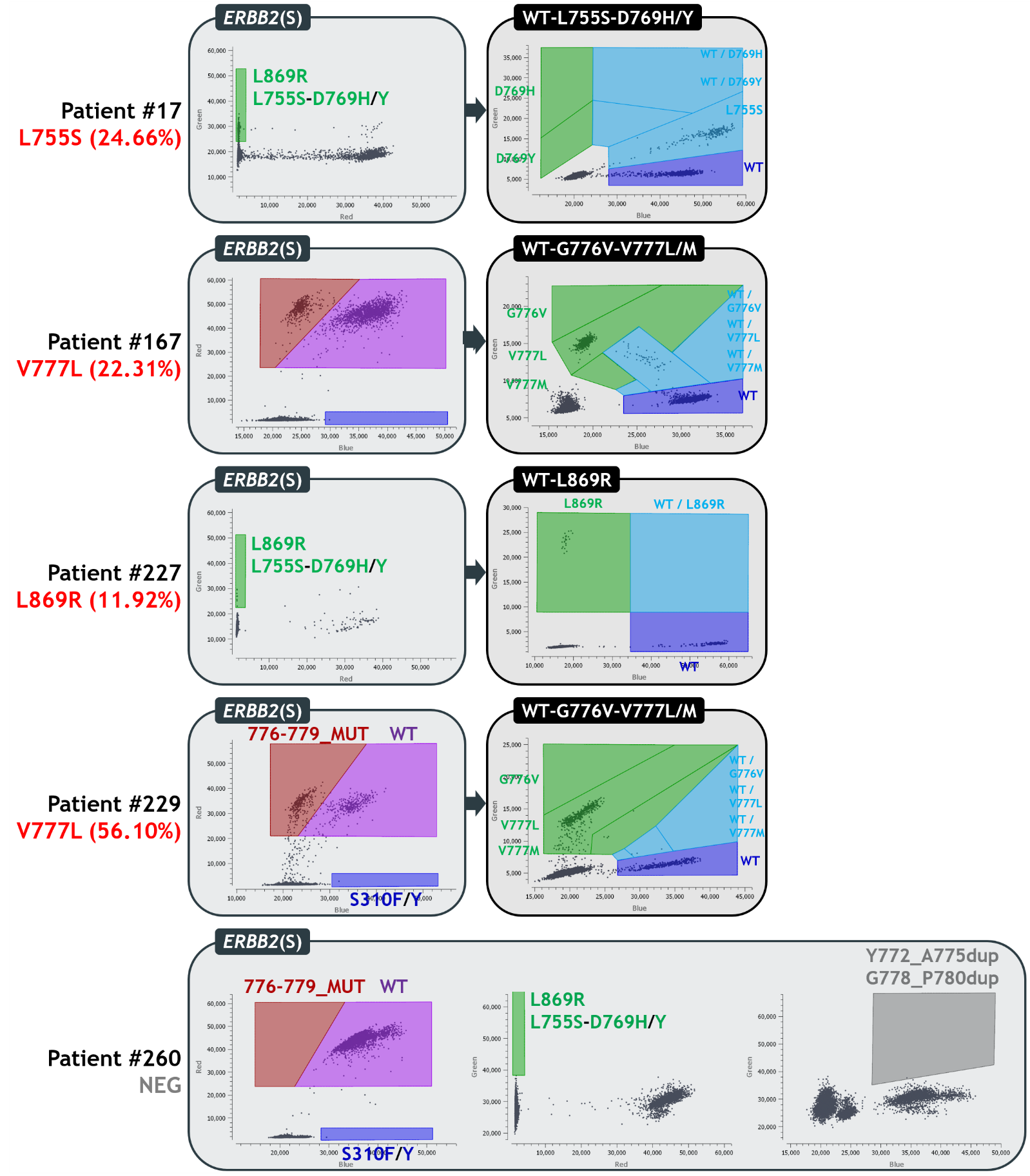
**

**Supplementary Figure S6. Results of the *ERBB2*(S) and WT-MUT Duplex assays performed on patient tumor tissue samples.** 2D dot plot results of *ERBB2* mutations (L755S, V777L, L869R, and V777L) first detected using the *ERBB2*(S) assay with the respective MAFs of 20.41%, 22.31%, 13.98%, and 50.48%. Results were confirmed using the corresponding WT-MUT Duplex assay with the respective MAFs of 24.66%, 22.76%, 11.92%, and 56.10%.

*(MAF: mutant allelic frequency).*

| **ITEM TO CHECK** | **PROVIDED** | **COMMENT** |
| --- | --- | --- |
| **Column1** | **Y/N** | **Column2** |
| **1. SPECIMEN** |  |  |
| Detailed description of specimen type and numbers | **Y** | Materials and Methods |
| Sampling procedure (including time to storage) | **Y** | Materials and Methods |
| Sample aliquotation, storage conditions, and duration | **Y** | Materials and Methods  Plasma samples were stored at -80°C in 5 ml cryotubes (Cryo.s^TM^, Greiner Bio-One Internationa, Kremsmünster, Austrial) for up to 47 months. |
| **2. NUCLEIC ACID EXTRACTION** |  |  |
| Description of extraction method, including amount of sample processed | **Y** | Materials and Methods |
| Volume of solvent used to elute/resuspend extract | **Y** | Materials and Methods |
| Number of extraction replicates | **Y** | All nucleic acid extractions were performed in simplicates. |
| Extraction blanks included? | **N** | Extraction blanks were only performed during the validation experiments of the extraction procedures and were then not included in the following extraction series for economic considerations. |
| **3. NUCLEIC ACID ASSESSMENT AND STORAGE** |  |  |
| Method to evaluate quality of nucleic acids | **Y** | The quality of nucleic acids was assessed using the High Sensitivity DNA kit on a 2100 Bioanalyzer Instrument (Agilent Technologies, Santa Clara, California, United States) during the validation experiments of the extraction procedures and was then not realized in the following extraction series for economic considerations. |
| Method to evaluate quantity of nucleic acids (including molecular weight and calculations when using mass) | **Y** | Materials and Methods |
| Storage conditions: temperature, concentration, duration, buffer, aliquots | **Y** | After quantification with the Qubit^TM^, each cfDNA sample was divided into three aliquots of 16 µl stored at -80°C for up to 39 months, in the elution buffer. |
| Clear description of dilution steps used to prepare working DNA solution | **Y** | Materials and Methods |
| **4. NUCLEIC ACID MODIFICATION** | **NA** | NA |
| Template modification (digestion, sonication, pre-amplification, bisulphite etc.) | **NA** | NA |
| Details of repurification following modification if performed | **NA** | NA |
| **5. REVERSE TRANSCRIPTION** | **NA** | All templates measured with dPCR were DNA templates. |
| cDNA priming method and concentration | **NA** | NA |
| One or two step protocol (include reaction details for two step) | **NA** | NA |
| Amount of RNA added per reaction | **NA** | NA |
| Detailed reaction components and conditions | **NA** | NA |
| Estimated copies measured with and without addition of RT | **NA** | NA |
| Manufacturer of reagents used with catalogue and lot numbers | **NA** | NA |
| Storage of cDNA: temperature, concentration, duration, buffer and aliquots | **NA** | NA |
| **6. dPCR OLIGONUCLEOTIDES DESIGN AND TARGET INFORMATION** |  |  |
| Sequence accession number or official gene symbol | **Y** | Materials and Methods |
| Method (software) used for design and *in silico* verification | **Y** | Materials and Methods |
| Location of amplicon | **Y** | Materials and Methods; Supplementary Table S2 |
| Amplicon length | **Y** | Supplementary Table S2 |
| Primer and probe sequences (or amplicon context sequence) | **Y** | Supplementary Table S2 |
| Location and identity of any modifications | **Y** | Supplementary Table S2 |
| Manufacturer of oligonucleotides | **Y** | Materials and Methods |
| **7. dPCR PROTOCOL** |  |  |
| Manufacturer of dPCR instrument and instrument model | **Y** | Materials and Methods |
| Buffer/kit manufacturer with catalogue and lot number | **Y** | Materials and Methods (cat. number: R10054; lot: #: Buffer A: MF21H26EGT1, Buffer B: D21J04EGT1, Stilla Technologies, Villejuif, France) |
| Primer and probe concentration | **Y** | Supplementary Table S2 |
| Pre-reaction volume and composition (incl. amount of template and if restriction enzyme added) | **Y** | Materials and Methods |
| Template treatment (initial heating or chemical denaturation) | **Y** | Materials and Methods |
| Polymerase identity and concentration, Mg++ and dNTP concentrations | **N** | Information not available for commercial disclosure reasons. |
| Complete thermocycling parameters | **Y** | Supplementary Table S5 |
| **8. ASSAY VALIDATION** |  |  |
| Details of optimisation performed | **Y** | Materials and Methods; Results; Figure 2 |
| Analytical specificity (vs. related sequences) and limit of blank (LOB) | **Y** | Supplementary Table S7 |
| Analytical sensitivity/LoD and how this was evaluated | **Y** | Supplementary Table S7 |
| Testing for inhibitors (from biological matrix/extraction) | **Y** | Was performed during the validation experiments; Data not shown. |
| **9. DATA ANALYSIS** |  |  |
| Description of dPCR experimental design | **Y** | Materials and Methods |
| Comprehensive details negative and positive of controls (whether applied for QC or for estimation of error) | **Y** | Materials and Methods |
| Partition classification method (thresholding) | **Y** | Materials and Methods; Results; Figure 2 |
| Examples of positive and negative experimental results (including fluorescence plots in supplementary material) | **Y** | Results; Figure 5; Supplementary Figure S4 |
| Description of technical replication | **Y** | Supplementary Figure S3 |
| Repeatability (intra-experiment variation) | **Y** | Supplementary Table S8 |
| Reproducibility (inter-experiment/user/lab etc. variation) | **Y** | Supplementary Figure S3 |
| Number of partitions measured (average and standard deviation) | **Y** | The data generated during the current study are available from the corresponding author upon request. |
| Partition volume | **Y** | 0.68 nl |
| Copies per partition (λ or equivalent) (average and standard deviation) | **Y** | The data generated during the current study are available from the corresponding author upon request. |
| dPCR analysis program (source, version) | **Y** | Materials and Methods |
| Description of normalisation method | **NA** | NA |
| Statistical methods used for analysis | **Y** | Materials and Methods |
| Data transparency | **Y** | The data generated during the current study are available from the corresponding author upon request. |

**Supplementary Table S1.** **Completed dMIQE2020 checklist.** *(Y: Yes; N: No; NA: not applicable)*

| ***ERBB2*(S) assay** | | | | | | |
| --- | --- | --- | --- | --- | --- | --- |
| **OLIGO**  **NAME** | **OLIGO TYPE** | **5’-FLUOROPHORE** | **SEQUENCE**  **(5’ – 3’)** | **3’-MODIFICATION** | **AMPLICON LENGTH (bp)** | **FINAL CONCENTRATION (µM)** |
| 310_Fwd | Primer | NA | AGACAACTACCTTTCTACGG | NA | 76 | 0.55 |
| 310_Rev | Primer | NA | CTCTGCTGTCACCTCTTG | NA |  | 0.55 |
| 755-769_Fwd | Primer | NA | ATGTGAAAATTCCAGTGGC | NA | 115 | 0.65 |
| 755-769_Rev | Primer | NA | TCCTTCCTGTCCTCCTAG | NA |  | 0.65 |
| 772-780_Fwd | Primer | NA | CTCCCATACCCTCTCAGC | NA | 105 | 0.75 |
| 772-780_Rev | Primer | NA | GTGGATGTCAGGCAGATG | NA |  | 0.75 |
| 869_Fwd | Primer | NA | CAAAATTACAGACTTCGGGC | NA | 59 | 0.6 |
| 869_Rev | Primer | NA | CATGGTACTCTGTCTCGTC | NA |  | 0.6 |
| S310F_Prb | Hydrolysis probe | FAM | TGGGATT+CTGCA+CCC | BHQ-1 | NA | 0.25 |
| S310Y_Prb | Hydrolysis probe | FAM | TGGGATA+CTGCA+CCC | BHQ-1 | NA | 0.25 |
| L755S_Prb | Hydrolysis probe | HEX | CAA+AGTGTCGA+G+GGA | BHQ-1 | NA | 0.35 |
| D769H_Prb | Hydrolysis probe | HEX | T+CTTA+CA+CGTAAG+CC | BHQ-1 | NA | 0.4 |
| D769Y_Prb | Hydrolysis probe | HEX | T+CTT+A+TA+CG+TAAG+CC | BHQ-1 | NA | 0.375 |
| Y772_A775dup_Prb | Hydrolysis probe | HEX | TGA+TGGCA+T+ACG+TGA | BHQ-1 | NA | 1 |
| DO[776-779]_Prb | Hydrolysis probe | FAM | TGGTGTGGGCTCCCC+ATA | BHQ-1 | NA | 0.9 |
| REF[776-779]_Prb | Hydrolysis probe | Cy5 | A+CCCTTGTCCCCAGGAA | BHQ-2 | NA | 0.775 |
| G778_P780dup_Prb | Hydrolysis probe | HEX | C+CNGGCTCCC+CAT+AT | BHQ-1 | NA | 0.125 |
| L869R_Prb | Hydrolysis probe | HEX | CTCGGC+GGCTGGACA | BHQ-1 | NA | 0.4 |
| WT_310__Blk | Non-fluorescent blocker | NA | TGGGATC+CTGCA+CCC | Phosphate | NA | 0.075 |

***(Part 1 of 3)***

| **WT-S310F/Y Duplex** | | | | | | |
| --- | --- | --- | --- | --- | --- | --- |
| **OLIGO**  **NAME** | **OLIGO TYPE** | **5’-FLUOROPHORE** | **SEQUENCE**  **(5’ – 3’)** | **3’-MODIFICATION** | **AMPLICON LENGTH (bp)** | **FINAL CONCENTRATION (µM)** |
| 310_Fwd | Primer | NA | AGACAACTACCTTTCTACGG | NA | 76 | 1 |
| 310_Rev | Primer | NA | CTCTGCTGTCACCTCTTG | NA |  | 1 |
| WT_310__Prb | Hydrolysis probe | HEX | T+GGG+ATCCTGCACCC | BHQ-1 | NA | 0.75 |
| S310F_Prb | Hydrolysis probe | FAM | TGGGATT+CTGCA+CCC | BHQ-1 | NA | 0.75 |
| S310Y_Prb | Hydrolysis probe | FAM | TGGGATA+CTGCA+CCC | BHQ-1 | NA | 0.3 |
| **WT-L755S-D769H/Y Duplex** | | | | | | |
| **OLIGO**  **NAME** | **OLIGO TYPE** | **5’-FLUOROPHORE** | **SEQUENCE**  **(5’ – 3’)** | **3’-MODIFICATION** | **AMPLICON LENGTH (bp)** | **FINAL CONCENTRATION (µM)** |
| 755-769_Fwd | Primer | NA | ATGTGAAAATTCCAGTGGC | NA | 115 | 1 |
| 755-769_Rev | Primer | NA | TCCTTCCTGTCCTCCTAG | NA |  | 1 |
| WT_769__Prb | Hydrolysis probe | FAM | T+CT+T+AGA+CGTAAGCC | BHQ-1 | NA | 0.5 |
| L755S_Prb | Hydrolysis probe | HEX | CAA+AGTGTCGA+G+GGA | BHQ-1 | NA | 0.25 |
| D769H_Prb | Hydrolysis probe | HEX | T+CTTA+CA+CGTAAG+CC | BHQ-1 | NA | 0.4 |
| D769Y_Prb | Hydrolysis probe | HEX | T+CTT+A+TA+CG+TAAG+CC | BHQ-1 | NA | 0.2 |
| **WT-G776V-V777L/M Duplex** | | | | | | |
| **OLIGO**  **NAME** | **OLIGO TYPE** | **5’-FLUOROPHORE** | **SEQUENCE**  **(5’ – 3’)** | **3’-MODIFICATION** | **AMPLICON LENGTH (bp)** | **FINAL CONCENTRATION (µM)** |
| 772-780_Fwd | Primer | NA | CTCCCATACCCTCTCAGC | NA | 105 | 1 |
| 772-780_Rev | Primer | NA | GTGGATGTCAGGCAGATG | NA |  | 1 |
| DO[776-779]_Prb | Hydrolysis probe | FAM | TGGTGTGGGCTCCCC+ATA | BHQ-1 | NA | 0.5 |
| G776V_Prb | Hydrolysis probe | HEX | ATGGCTGTTGT+G+GGC | BHQ-1 | NA | 0.75 |
| V777L_Prb | Hydrolysis probe | HEX | TGGC+TGGTYT+GGGCT | BHQ-1 | NA | 0.5 |
| V777M_Prb | Hydrolysis probe | HEX | TGG+CTGGTA+TGGGCT | BHQ-1 | NA | 0.075 |
| **WT-ins Duplex** | | | | | | |
| **OLIGO**  **NAME** | **OLIGO TYPE** | **5’-FLUOROPHORE** | **SEQUENCE**  **(5’ – 3’)** | **3’-MODIFICATION** | **AMPLICON LENGTH (bp)** | **FINAL CONCENTRATION (µM)** |
| 772-780_Fwd | Primer | NA | CTCCCATACCCTCTCAGC | NA | 105 | 1 |
| 772-780_Rev | Primer | NA | GTGGATGTCAGGCAGATG | NA |  | 1 |
| DO[776-779]_Prb | Hydrolysis probe | FAM | TGGTGTGGGCTCCCC+ATA | BHQ-1 | NA | 0.5 |
| G778_S779insLPG_Prb | Hydrolysis probe | HEX | TGGGGCTCCCCGGCT | BHQ-1 | NA | 0.25 |
| G778_S779insLPS_Prb | Hydrolysis probe | HEX | TGGGGC+TCCCCAGCT | BHQ-1 | NA | 1 |
| **WT-dup Duplex** | | | | | | |
| **OLIGO**  **NAME** | **OLIGO TYPE** | **5’-FLUOROPHORE** | **SEQUENCE**  **(5’ – 3’)** | **3’-MODIFICATION** | **AMPLICON LENGTH (bp)** | **FINAL CONCENTRATION (µM)** |
| 772-780_Fwd | Primer | NA | CTCCCATACCCTCTCAGC | NA | 105 | 1 |
| 772-780_Rev | Primer | NA | GTGGATGTCAGGCAGATG | NA |  | 1 |
| DO[776-779]_Prb | Hydrolysis probe | FAM | TGGTGTGGGCTCCCC+ATA | BHQ-1 | NA | 0.5 |
| Y772_A775dup_Prb | Hydrolysis probe | HEX | TGA+TGGCA+T+ACG+TGA | BHQ-1 | NA | 0.75 |
| G778_P780dup_Prb | Hydrolysis probe | HEX | C+CNGGCTCCC+CAT+AT | BHQ-1 | NA | 0.25 |

***(Part 2 of 3)***

| **WT-L869R Duplex** | | | | | | |
| --- | --- | --- | --- | --- | --- | --- |
| **OLIGO**  **NAME** | **OLIGO TYPE** | **5’-FLUOROPHORE** | **SEQUENCE**  **(5’ – 3’)** | **3’-MODIFICATION** | **AMPLICON LENGTH (bp)** | **FINAL CONCENTRATION (µM)** |
| 869_Fwd | Primer | NA | CAAAATTACAGACTTCGGGC | NA | 59 | 1 |
| 869_Rev | Primer | NA | CATGGTACTCTGTCTCGTC | NA |  | 1 |
| WT_869__Prb | Hydrolysis probe | FAM | CTCG+GC+TGCTGGACA | BHQ-1 | NA | 0.5 |
| L869R_Prb | Hydrolysis probe | HEX | CTCGGC+GGCTGGACA | BHQ-1 | NA | 0.5 |

***(Part 3 of 3)***

**Supplementary Table S2.** **Oligonucleotides composing the *ERBB2* assays.** List of all the oligonucleotides (oligos) composing the *ERBB2* assays (primers, hydrolysis probes, and non-fluorescent blockers), with amplicon length provided for each primer pair, as well as 5’- and 3’-modifications for probes and blockers and the final concentrations (µM) for each oligo.

*(NA: not applicable; +N: locked nucleic acid (LNA); FAM: Carboxyfluorescein; Fwd: forward; HEX: Hexachlorofluorescein; Rev: reverse; Prb: probe; BHQ: black hole quencher).*

| **Mutations detected by the *ERBB2*(S) assay** | | | | | | |
| --- | --- | --- | --- | --- | --- | --- |
| **MUTATION** | | **COSMIC**  **MUTATION ID** | **GENOMIC COORDINATES** | **COSMIC**  **COUNT** | **COSMIC COUNT**  **FREQUENCY (%)** |  |
| **CDS** | **AA** |  |  |  |  |  |
| c.2264T>C | p.L755S | COSM14060 | GRCh38, 17:39723967..39723967 | 114 | 19.55 |  |
| c.2329G>T | p.V777L | COSM14062 | GRCh38, 17:39724747..39724747 | 55 | 9.43 |  |
| c.929C>T | p.S310F | COSM48358 | GRCh38, 17:39711955..39711955 | 30 | 5.15 |  |
| c.2305G>T | p.D769Y | COSM1251412 | GRCh38, 17:39724008..39724008 | 28 | 4.80 |  |
| c.929C>A | p.S310Y | COSM94225 | GRCh38, 17:39711955..39711955 | 17 | 2.92 |  |
| *c.2313_2324dup* | *p.Y772_A775dup* | COSM20959 | GRCh38, 17:39724742..39724743 | 16 | 2.74 |  |
| c.2329G>C | p.V777L | COSM436500 | GRCh38, 17:39724747..39724747 | 16 | 2.74 |  |
| *c.2332_2340dup* | *p.G778_P780dup* | COSM12556 | GRCh38, 17:39724758..39724759 | 16 | 2.74 |  |
| c.2606T>G | p.L869R | COSM249793 | GRCh38, 17:39725161..39725161 | 14 | 2.40 |  |
| *c.2331_2339dup* | *p.G778_P780dup* | COSM12555 | GRCh38, 17:39724757..39724758 | 10 | 1.72 |  |
| c.2305G>C | p.D769H | COSM13170 | GRCh38, 17:39724008..39724008 | 6 | 1.03 |  |
| *c.2333_2334insGCTCCC...* | *p.G778_S779insLPG* | COSM7449770 | GRCh38, 17:39724751..39724752 | 6 | 1.03 |  |
| *c.2333_2334insGCTCCC...* | *p.G778_S779insLPS* | COSM5802314 | GRCh38, 17:39724751..39724752 | 6 | 1.03 |  |
| c.2327G>T | p.G776V | COSM18609 | GRCh38, 17:39724745..39724745 | 3 | 0.51 |  |
| c.2329G>A | p.V777M | COSM14064 | GRCh38, 17:39724747..39724747 | 1 | 0.17 |  |
| *c.2339_2340insCGGCTC...* | *p.G778_P780dup* | COSM6865893 | GRCh38, 17:39724757..39724758 | 1 | 0.17 |  |
| *c.2339_2340insTGGCTC...* | *p.G778_P780dup* | COSM303948 | GRCh38, 17:39724757..39724758 | 1 | 0.17 |  |

**Supplementary Table S3.** **Mutations detected by the *ERBB2*(S) assay.** List of all the pathogenic (in black) and non-pathogenic (in blue and italics) *ERBB2* mutations detected by the *ERBB2*(S) assay. Categorization was according to the information provided in the Catalogue of Somatic Mutations in Cancer (COSMIC) database for breast carcinoma tumor samples. The table includes coding sequences (CDS), amino acid (AA) characteristics, legacy mutation identifiers (ID), genomic coordinates on the genome reference consortium human build 38 (GRCh38), count, and count frequency (%) calculated among all of the pathogenic mutations.

| **gBlock NAME** | **SEQUENCE**  **(5’ – 3’)** | **LENGTH**  **(bp)** |
| --- | --- | --- |
| S310F_gBlock | TGCACGAAGGGCCAGGGTATGTGGCTACATGTTCCTGATCTCCTTAGACAACTACCTTTCTACGGACGTGGGATTCTGCACCCTCGTCTGCCCCCTGCACAACCAAGAGGTGACAGCAGAGGATGGAACACAGCGGTGTGAGAAGTGCAGCAAGCCCTGTGCCCGA | 166 |
| S310Y_gBlock | TGCACGAAGGGCCAGGGTATGTGGCTACATGTTCCTGATCTCCTTAGACAACTACCTTTCTACGGACGTGGGATACTGCACCCTCGTCTGCCCCCTGCACAACCAAGAGGTGACAGCAGAGGATGGAACACAGCGGTGTGAGAAGTGCAGCAAGCCCTGTGCCCGA | 166 |
| L755S_gBlock | GGGCATCTGGATCCCTGATGGGGAGAATGTGAAAATTCCAGTGGCCATCAAAGTGTCGAGGGAAAACACATCCCCCAAAGCCAACAAAGAAATCTTAGACGTAAGCCCCTCCACCCTCTCCTGCTAGGAGGACAGGAAGGACCCCATGGCTGCAGGTCTGGGCTCT | 166 |
| D769H_gBlock | GGGCATCTGGATCCCTGATGGGGAGAATGTGAAAATTCCAGTGGCCATCAAAGTGTTGAGGGAAAACACATCCCCCAAAGCCAACAAAGAAATCTTACACGTAAGCCCCTCCACCCTCTCCTGCTAGGAGGACAGGAAGGACCCCATGGCTGCAGGTCTGGGCTCT | 166 |
| D769Y_gBlock | GGGCATCTGGATCCCTGATGGGGAGAATGTGAAAATTCCAGTGGCCATCAAAGTGTTGAGGGAAAACACATCCCCCAAAGCCAACAAAGAAATCTTATACGTAAGCCCCTCCACCCTCTCCTGCTAGGAGGACAGGAAGGACCCCATGGCTGCAGGTCTGGGCTCT | 166 |
| Y772_A775dup_gBlock | GTTGGGAGGCTGTGTGGTGTTTGGGGGTGTGTGGTCTCCCATACCCTCTCAGCGTACCCTTGTCCCCAGGAAGCATACGTGATGGCATACGTGATGGCTGGTGTGGGCTCCCCATATGTCTCCCGCCTTCTGGGCATCTGCCTGACATCCACGGTGCAGCTGGTGA | 166 |
| G776V_gBlock | GTGATGGTTGGGAGGCTGTGTGGTGTTTGGGGGTGTGTGGTCTCCCATACCCTCTCAGCGTACCCTTGTCCCCAGGAAGCATACGTGATGGCTGTTGTGGGCTCCCCATATGTCTCCCGCCTTCTGGGCATCTGCCTGACATCCACGGTGCAGCTGGTGACACAGC | 166 |
| V777L_G>C__gBlock | GTGATGGTTGGGAGGCTGTGTGGTGTTTGGGGGTGTGTGGTCTCCCATACCCTCTCAGCGTACCCTTGTCCCCAGGAAGCATACGTGATGGCTGGTCTGGGCTCCCCATATGTCTCCCGCCTTCTGGGCATCTGCCTGACATCCACGGTGCAGCTGGTGACACAGC | 166 |
| V777L_G>T__gBlock | GTGATGGTTGGGAGGCTGTGTGGTGTTTGGGGGTGTGTGGTCTCCCATACCCTCTCAGCGTACCCTTGTCCCCAGGAAGCATACGTGATGGCTGGTTTGGGCTCCCCATATGTCTCCCGCCTTCTGGGCATCTGCCTGACATCCACGGTGCAGCTGGTGACACAGC | 166 |
| V777M_gBlock | GTGATGGTTGGGAGGCTGTGTGGTGTTTGGGGGTGTGTGGTCTCCCATACCCTCTCAGCGTACCCTTGTCCCCAGGAAGCATACGTGATGGCTGGTATGGGCTCCCCATATGTCTCCCGCCTTCTGGGCATCTGCCTGACATCCACGGTGCAGCTGGTGACACAGC | 166 |
| G778_P780dup_gBlock(1) | GGTTGGGAGGCTGTGTGGTGTTTGGGGGTGTGTGGTCTCCCATACCCTCTCAGCGTACCCTTGTCCCCAGGAAGCATACGTGATGGCTGGTGTGGGCTCCCCGGGCTCCCCATATGTCTCCCGCCTTCTGGGCATCTGCCTGACATCCACGGTGCAGCTGGTGACA | 166 |
| G778_P780dup_gBlock(2) | GGTTGGGAGGCTGTGTGGTGTTTGGGGGTGTGTGGTCTCCCATACCCTCTCAGCGTACCCTTGTCCCCAGGAAGCATACGTGATGGCTGGTGTGGGCTCCCCAGGCTCCCCATATGTCTCCCGCCTTCTGGGCATCTGCCTGACATCCACGGTGCAGCTGGTGACA | 166 |
| G778_S779insLPG_gBlock | GGTTGGGAGGCTGTGTGGTGTTTGGGGGTGTGTGGTCTCCCATACCCTCTCAGCGTACCCTTGTCCCCAGGAAGCATACGTGATGGCTGGTGTGGGGCTCCCCGGCTCCCCATATGTCTCCCGCCTTCTGGGCATCTGCCTGACATCCACGGTGCAGCTGGTGACA | 166 |
| G778_S779insLPS_gBlock | GGTTGGGAGGCTGTGTGGTGTTTGGGGGTGTGTGGTCTCCCATACCCTCTCAGCGTACCCTTGTCCCCAGGAAGCATACGTGATGGCTGGTGTGGGGCTCCCCAGCTCCCCATATGTCTCCCGCCTTCTGGGCATCTGCCTGACATCCACGGTGCAGCTGGTGACA | 166 |
| L869R_gBlock | CGTACACAGGGACTTGGCCGCTCGGAACGTGCTGGTCAAGAGTCCCAACCATGTCAAAATTACAGACTTCGGGCTGGCTCGGCGGCTGGACATTGACGAGACAGAGTACCATGCAGATGGGGGCAAGGTTAGGTGAAGGACCAAGGAGCAGAGGAGGCTGGGTGGA | 166 |

**Supplementary Table S4.** **Mutated gBlocks used in this study.** List of the 15 mutated gBlocks used in this study and their nucleotide sequence. All gBlocks were designed to measure 166 bp to mimic the average cfDNA length. Mutated nucleotides are identified in red, and inserted nucleotides are identified in light blue.

| **PCR Program** | |
| --- | --- |
| **STEP** | **DETAILS** |
| **1** | Partition at 40°C, "Sapphire V1" |
| **2** | Temp. 95.0°C for 10' 0" Wait |
| **3** | Start Cycle, 45x |
| **4** | Temp. 95.0°C for 30" Wait |
| **5** | Temp. 58.0°C for 30" Wait |
| **6** | Close Cycle |
| **7** | Release P, "Sapphire V1" |

**Supplementary Table S5. PCR program used for the *ERBB2* assays.** Detailed steps of the PCR program used for the *ERBB2*(S) and WT-MUT Duplex assays on the naica^®^ Geode. The crystals of droplets were first generated during the ‘partition’ step (STEP 1) with an increase of pressure up to +1,000 mbar at a fixed temperature of 40°C. STEP 1 was followed by the PCR amplification steps (STEP 2 to 6) and the final release step (STEP 7) to return to ambient temperature and pressure conditions.

| **Scanning parameters** | |
| --- | --- |
| **PARAMETER** | **VALUE** |
| **FOCUS** | 0.74 mm |
| **FAM** | 90 ms |
| **HEX** | 100 ms |
| **Cy5** | 100 ms |

**Supplementary Table S6. Scanning parameters applied for the *ERBB2* assays.** Detailed values of the different parameters applied during the scanning of the Sapphire chips on the naica^®^ Prism3 used for the *ERBB2*(S) and WT-MUT Duplex assays.

*(Cy5: Cyanine-5; FAM: Carboxyfluorescein; HEX: Hexachlorofluorescein).*

| **LOB_95%_ and theoretical LOD_95%_** | | | | | | | |
| --- | --- | --- | --- | --- | --- | --- | --- |
| **MUTATION DETECTION** | **NUMBER OF REPLICATES** | **LOB_95%_** | **LOD_95%_** | | | | |
|  |  | **droplets** | **droplets** | **copies/µl of PCR Mix** | **copies/PCR** | **copies/ml of plasma** | **MAF (%)** |
| S310F/Y | 1 | 3 | 8 | 0.579 | 14.5 | 9.7 | 0.145 |
|  | 2 | 4 | 9 | 0.344 | 8.6 | 5.7 | 0.086 |
|  | 3 | 5 | 11 | 0.264 | 6.6 | 4.4 | 0.066 |
| L755S-D769H/Y-L869R | 1 | 4 | 9 | 0.688 | 17.2 | 11.5 | 0.172 |
|  | 2 | 6 | 12 | 0.448 | 11.2 | 7.5 | 0.112 |
|  | 3 | 8 | 15 | 0.365 | 9.1 | 6.1 | 0.091 |
| Y772_A775dup-G778_P780dup | 1 | 4 | 9 | 0.688 | 17.2 | 11.5 | 0.172 |
|  | 2 | 5 | 11 | 0.396 | 9.9 | 6.6 | 0.099 |
|  | 3 | 6 | 12 | 0.298 | 7.5 | 5.0 | 0.075 |
| 776-779_MUT_ | 1 | 5 | 11 | 0.793 | 19.8 | 13.2 | 0.198 |
|  | 2 | 8 | 15 | 0.548 | 13.7 | 9.1 | 0.137 |
|  | 3 | 10 | 17 | 0.430 | 10.8 | 7.2 | 0.108 |

**Supplementary Table S7.** **LOB_95%_ and theoretical LOD_95%_ values for the *ERBB2*(S) assay.** The limit of blank (LOB_95%_), defined as the maximum number of false-positive droplets expected in a chamber with a probability of 95% (i.e., α risk equal to 5%) in a sample containing no target sequence, and the theoretical limit of detection (LOD_95%_), defined as the minimum concentration that can be said to be non-zero and statistically higher than the limit of blank, with a probability of 95%, were determined as previously described [22,23]. The LOD_95%_ is expressed either in droplets or in copies/µl of PCR Mix, the latter of which can be converted into copies/PCR (for 25 µl of PCR Mix per replicate), copies/ml of plasma (for 15 µl of input DNA, 50 µl of elution volume, and 5 ml of plasma), or MAF (%) (for our maximum concentration of 10,000 copies of total cfDNA per replicate). However, these values are theoretical, as plasma volumes and cfDNA concentration vary for each patient.

| **Repeatability study results** | | | | |
| --- | --- | --- | --- | --- |
| **DETECTION** | **EXPECTED CONCENTRATIONS (copies/PCR)** | **MEASURED CONCENTRATIONS**  **(copies/PCR)** | | |
|  |  | **MEAN** | **SD** | **CV (%)** |
| WT | 1,000 | 888 | 110.6 | 12.5 |
| S310F/Y | 1,000 | 1,310 | 88.7 | 6.8 |
| L755S-D769H/Y-L869R | 1,000 | 1,291 | 44.2 | 3.4 |
| Y772_A775dup-G778_P780dup | 1,000 | 1,558 | 68.6 | 4.4 |
| 776-779_MUT_ | 1,000 | 1,129 | 62.1 | 5.5 |

**Supplementary Table S8.** **Results obtained during the repeatability study for the *ERBB2*(S) assay.** A DNA mixture with a theoretical concentration of 1,000 copies/PCR for each detection was prepared. Each DNA mixture was tested in twelve replicates during the same PCR experiments in order to study the repeatability of the measures; this was done by calculating the coefficient of variation (CV) using the following equation: $CV (\%)=\frac{\sigma}{\mu}\times100$, where $\sigma$ is the standard deviation and $\mu$ the mean of the replicate results.

*Please note that, due to their short length (166 bp), the mass represented by one copy of a mutated gBlock is extremely low (1.7.10^-10^ ng). Therefore, in order to reach the concentrations required to prepare these DNA mixtures, each mutated gBlock stock solution must be diluted 1:1,000,000 via a cascade dilution strategy, as stock solutions are concentrated to 10 ng/µl, which is 5.9.10^10^ copies/µl. Therefore, it is not abnormal to observe marked differences between the ‘expected values’ and the ‘measured values’.*

|  | **COSMIC Database** | | **Results obtained on our series of plasma samples** | |
| --- | --- | --- | --- | --- |
| **MUTATION**  **(AA)** | **NUMBER OF MUTATIONS** | **FREQUENCY**  **(%)** | **NUMBER OF MUTATIONS** | **FREQUENCY**  **(%)** |
| L755S | 114 | 19.55 | 3 | 25.00 |
| V777L | 55 | 9.43 | 3 | 25.00 |
| S310F | 30 | 5.15 | 1 | 8.33 |
| S310Y | 17 | 2.92 | 2 | 16.67 |
| L869R | 14 | 2.40 | 2 | 16.67 |
| D769H | 6 | 1.03 | 1 | 8.33 |

**Supplementary Table S9. Comparison of mutation frequencies observed in the COSMIC database and our series of plasma samples.** *ERBB2* mutations are ranked in descending order of frequency according to COSMIC data. The frequencies displayed for the results on our series of plasma samples only take into account mutations detected and characterized by our assays.
